# Diversity in ATP concentrations in a single bacterial cell population revealed by quantitative single-cell imaging

**DOI:** 10.1038/srep06522

**Published:** 2014-10-06

**Authors:** Hideyuki Yaginuma, Shinnosuke Kawai, Kazuhito V. Tabata, Keisuke Tomiyama, Akira Kakizuka, Tamiki Komatsuzaki, Hiroyuki Noji, Hiromi Imamura

**Affiliations:** 1 Graduate School of Engineering, the University of Tokyo, Japan; 2 Graduate School of Frontier Biosciences, Osaka University, Japan; 3 CREST, JST, Japan; 4 QBiC, RIKEN, Japan; 5 Research Institute for Electronic Science, Hokkaido University, Japan; 6 Faculty of Science, Shizuoka University; 7 PRESTO, JST, Japan; 8 Graduate School of Biostudies, Kyoto University, Japan; 9 The Hakubi Center for Advanced Research, Kyoto University, Japan

## Abstract

Recent advances in quantitative single-cell analysis revealed large diversity in gene expression levels between individual cells, which could affect the physiology and/or fate of each cell. In contrast, for most metabolites, the concentrations were only measureable as ensemble averages of many cells. In living cells, adenosine triphosphate (ATP) is a critically important metabolite that powers many intracellular reactions. Quantitative measurement of the absolute ATP concentration in individual cells has not been achieved because of the lack of reliable methods. In this study, we developed a new genetically-encoded ratiometric fluorescent ATP indicator “QUEEN”, which is composed of a single circularly-permuted fluorescent protein and a bacterial ATP binding protein. Unlike previous FRET-based indicators, QUEEN was apparently insensitive to bacteria growth rate changes. Importantly, intracellular ATP concentrations of numbers of bacterial cells calculated from QUEEN fluorescence were almost equal to those from firefly luciferase assay. Thus, QUEEN is suitable for quantifying the absolute ATP concentration inside bacteria cells. Finally, we found that, even for a genetically-identical *Escherichia coli* cell population, absolute concentrations of intracellular ATP were significantly diverse between individual cells from the same culture, by imaging QUEEN signals from single cells.

Recent studies revealed that protein copy number is affected by the stochastic intrinsic noise in the gene expression system^1^,^2^. Thus, even in genetically uniform bacterial populations, the protein copy number in single cells is surprisingly diverse. Switching of bacterial cells to certain states, such as persister and sporulation, as well as high expression of the lac operon are thought to be controlled by stochastic events^3^,^4^,^5^,^6^, to ensure that only some cells in the population enter a particular state.

Because of the large variations in intracellular protein copy number, we hypothesized that intracellular energy levels may also vary between cells. However, no reliable evaluation method is available to test this hypothesis. The concentrations of most metabolites were only measured as the ensemble average of many cells^7^, and the extent of diversity of single-cell metabolite concentrations in the same population remained unknown. One of the best indices of cell energy level is the concentration of intracellular adenosine triphosphate (ATP), which determines the chemical equilibrium or reaction rate of various intracellular reactions. In this study, we investigated the diversity of absolute ATP concentrations in single bacterial cells. Several methods for monitoring intracellular ATP have been reported^8^,^9^,^10^,^11^,^12^, but many are not applicable to single bacterial cells and/or cannot be used to determine absolute ATP concentration. We have previously reported Förster resonance energy transfer (FRET)-type ATP biosensors (dubbed ‘ATeam') that respond to single-cell ATP concentrations^13^,^14^,^15^. However, such biosensors composed of 2 fluorescent proteins (FPs) are still limited in their quantitative capacity, because the maturation time lag of 2 FPs may result in sensors with immature acceptors and intracellular sensor degradation could produce degradation intermediates with separate donors and acceptors. These defective-but-fluorescent FRET-type sensors may result in bias of the overall signal. Since the fraction of such malfunctioning sensors is related to cell growth rate, growth rate change of the cell may cause undesired shifts in the signal (see the ATeam results below). This effect is particularly problematic in bacteria, as their growth rates span a wide range from rapid division to nearly no growth.

If the ATP biosensor possessed only one FP, the effects of maturation time lag and degradation of the sensor in the cell could be eliminated. Therefore, we developed a new single FP-based biosensor for measuring absolute ATP concentrations. This new sensor was found to be essentially insensitive to alternation in growth rate. We then used this sensor to quantify ATP concentrations of individual cells in order to examine the metabolic diversity within a single cultural population.

## Results

### Design of the new single FP-based ATP indicator “QUEEN”

**Figure 1:**
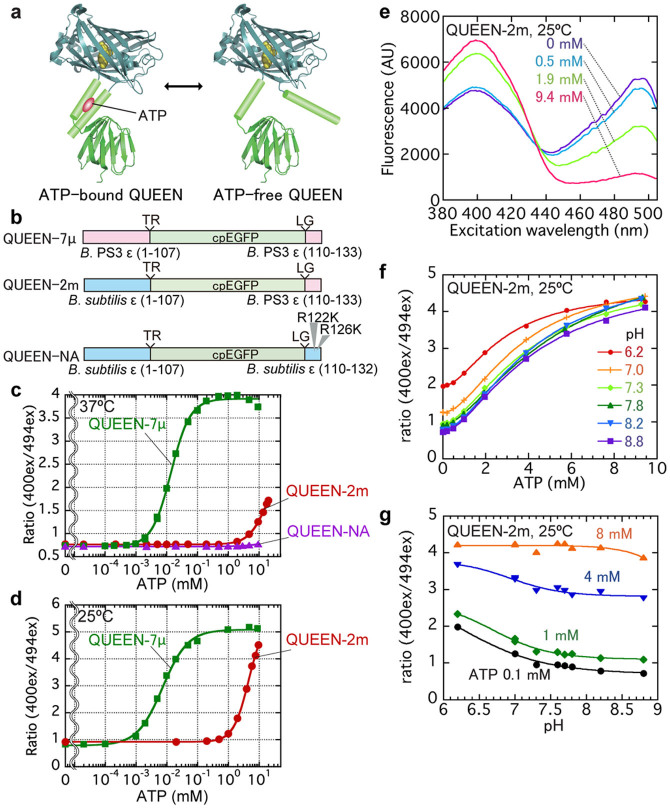
The developed single fluorescent protein (FP)-type ATP sensor (‘QUEEN') and its evaluation. (a) Schematic illustration of the ATP sensing mechanism of QUEEN. (b) Schematic illustration of the genetic structure of QUEENs. (c, d) 400ex/494ex ratio of purified QUEEN versus ATP at 25°C (c) and 37°C (d). (e) Excitation spectra of QUEEN-2m at various ATP concentrations. (f, g) Response of QUEEN-2m to ATP concentration at different pH values. The buffer composition was the same as buffer C, except for different buffer reagents (MES for pH 6.2 and 7.0; HEPES for pH 7.0–7.8; Tricine for pH 8.2 and 8.6).

Inspired by the Ca^2+^ biosensor PeriCam^16^ and G-CaMP^17^, circularly-permuted enhanced green fluorescent protein (cpEGFP)^18^ was inserted between 2 α-helices of the bacterial F_o_F_1_-ATP synthase ε subunit (Fig. 1a, b, also see Supplementary Note for details). 2-Amino acid (a.a.) linkers were inserted at each joint region. We named this series of biosensors “QUEEN” (for quantitative evaluator of cellular energy).

Three QUEEN variants (QUEEN-7µ, 2m and NA) are introduced in this study. QUEEN-7µ, which is based on the ε subunit of thermophilic *Bacillus* PS3, has the highest affinity to ATP (*K*_d_ ~ 7 µM at 25°C). QUEEN-2m is a QUEEN-7µ variant with its sequence partially exchanged with the ε subunit of *Bacillus subtilis*. QUEEN-2m was designed as a low affinity ATP indicator so it is suitable for measuring physiological ATP levels (*K*_d_ ~2 mM at 25°C). QUEEN-NA (for “no affinity”) is based on the ε subunit of *B. subtilis* and carries mutations in the ATP binding region^13^,^19^. QUEEN-NA is an ATP-insensitive version of QUEEN and thus was used in control experiments.

### In vitro characterization of QUEENs

The in vitro excitation spectra of QUEENs measured by emission intensity at 513 nm peaked at excitation wavelength of 400 nm and 494 nm (for an example, see Fig. 1e). The ratio of 513 nm emission intensity at these 2 excitation wavelengths, denoted 400ex/494ex hereafter, changed in response to ATP concentration. At 37°C, the *K*_d_ of QUEEN-7µ to ATP was 1.4 × 10^−2^ mM (Fig. 1c). QUEEN-7µ clearly showed selectivity against ATP rather than adenosine diphosphate (ADP) (see Supplementary Note and Supplementary Fig. S1b for details) At 25°C, its affinity to ATP was even higher (*K*_d_ = 7.2 × 10^−3^ mM) (Fig. 1d). However, QUEEN-7µ's affinity to ATP was too high for use in living bacteria cells. In a similar experiment, QUEEN-NA was confirmed that it does not respond to varying ATP concentrations (Fig 1c).

QUEEN-2m showed a *K*_d_ of 4.5 mM for ATP at 25°C, which is within the physiological ATP range (Supplementary Note). The 400ex/494ex ratio of QUEEN-2m increased upon addition of ATP (Fig. 1c–e). We noticed that high concentrations of ADP negatively biases the signal, but the effect is relatively small (approximately 10% or less at physiological ADP concentrations) (Supplementary Note, Supplementary Fig. S1). In addition, the QUEEN-2m signal was nearly unaffected at pH 7.3–8.8 (Fig. 1f, g), and also by physiological Mg^2+^ concentrations in the 1–2 mM range^20^ (Supplementary Fig. S2).

### Sensitivity to cell growth rate change of FRET-based and single FP-based ATP indicators

**Figure 2:**
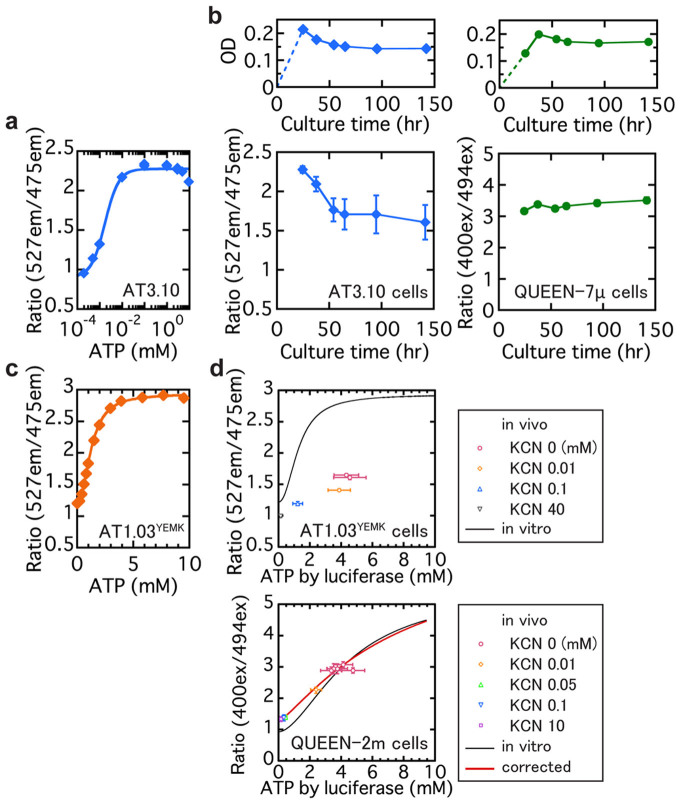
The comparison of single FP-type and FRET-type ATP indicators in vivo. (a) Response of the FRET-type AT3.10 sensor^13^ to ATP at 25°C in vitro. Measurement was performed in buffer C. *K*_d_ = 1.8 × 10^−3^ mM. (b) Time courses of in vivo QUEEN-7µ and AT3.10 signals in cells grown in continuous culture (25°C). (c) Response of FRET-type AT1.03^YEMK^ sensor^13^ to ATP at 37°C in vitro. Measurement was performed in buffer B. *K*_d_ = 1.3 mM. (d) In vivo AT1.03^YEMK^ (37°C) and QUEEN-2m (25°C) cell suspension measurement results compared to in vitro measurements. Cell suspension was treated by KCN of various concentrations to change the intracellular ATP concentrations. Different markers indicate the different concentration of KCN used. The horizontal axis is the intracellular ATP value quantified by luciferase assay. The solid black lines indicate the expected values from the purified ATeam/QUEEN measurements. The solid red line indicates the response of QUEEN-2m in vivo after correction. Error bars indicate standard errors (see Supplementary Methods).

We next tested whether the influence of maturation or degradation kinetics on the signal is reduced in QUEENs compared to ATeam. We cultured *E. coli* cells expressing AT3.10 (a variant of ATeam analogous to QUEEN-7µ in having a high affinity to ATP) or QUEEN-7µ in a continuous culture system. These indicators are insensitive to physiological ATP concentrations (1–10 mM; Fig 1d, 2a). If maturation or degradation kinetics affects the signaling of sensors, the signals of these indicators should fluctuate not with changes in ATP concentration, but with drastic changes in growth rate (Supplementary Note). Indeed, after the optical density (OD) reached ~0.2 and the growth rate became limited by the dilution rate of the continuous culture (0.10–0.12 h^−1^), the signal of AT3.10 cells (ratio of emission intensity at 527 nm and 475 nm upon excitation at 435 nm; 527em/475em) fluctuated. In contrast, the signal of QUEEN-7µ was virtually the same throughout the culture period (Fig. 2b). This insensitivity to the growth rate indicates that QUEEN biosensor signals are virtually unaffected by maturation or degradation kinetics (Supplementary Note).

### In vivo quantitative capacity of FRET-based and single FP-based ATP indicators

Next, to investigate the quantitative capacity of the indicators in vivo, we measured the fluorescence of an *E. coli* cell suspension expressing fluorescent ATP indicators. The indicators used in this experiment were either AT1.03^YEMK^, a variation of ATeam which is analogous to QUEEN-2m in having a low-millimolar affinity for ATP (Fig. 2c), or QUEEN-2m. To produce cells with varying intracellular concentrations, we varied the concentration of the respiration inhibitor potassium cyanide (KCN). The fluorescence of the cells were measured first, and then portions of the same culture were analyzed using a luciferase ATP assay to estimate the actual ATP concentration. The obtained result was compared to the in vitro ATP response curves (Fig. 2d). The signal of in vivo AT1.03^YEMK^ was always much lower than that in vitro. In contrast, the 400ex/494ex signal of in vivo QUEEN-2m was much more similar to the in vitro results. The slight deviation in the low ATP regions in Figure 2d was calibrated in the later measurement using the data obtained here (See Supplementary Methods). Additionally, the in vivo dynamic range of QUEEN-2m (~3.0) (Supplementary Note) was larger than that of ATeam1.03^YEMK^ (~1.8). Therefore, QUEEN showed a better quantitative capacity compared to ATeam, particularly in bacteria that have wide ranges of growth rates. It is not clear why in vivo QUEEN signals do not perfectly match the in vitro signals. The buffer condition we use in the in vitro measurement may not perfectly mimic intracellular condition.

### Evaluation of the precision of the single-cell ATP measurement by QUEEN

To calculate the ATP concentration from single cell QUEEN-2m signal, the error included in the experimental system should be considered in advance. For this purpose, we first recorded the images of QUEEN-7µ and QUEEN-NA. The signal of QUEEN-7µ is considered to be saturated at physiological (mM) ATP concentrations (Fig. 1d). QUEEN-NA is the QUEEN variant which does not bind ATP (Fig. 1c). Therefore, the signals of QUEEN-7µ or QUEEN-NA cells should be the same for all cells regardless of ATP level. The distribution of the signal obtained for these variants reflects the noise of the experimental system.

**Figure 3:**
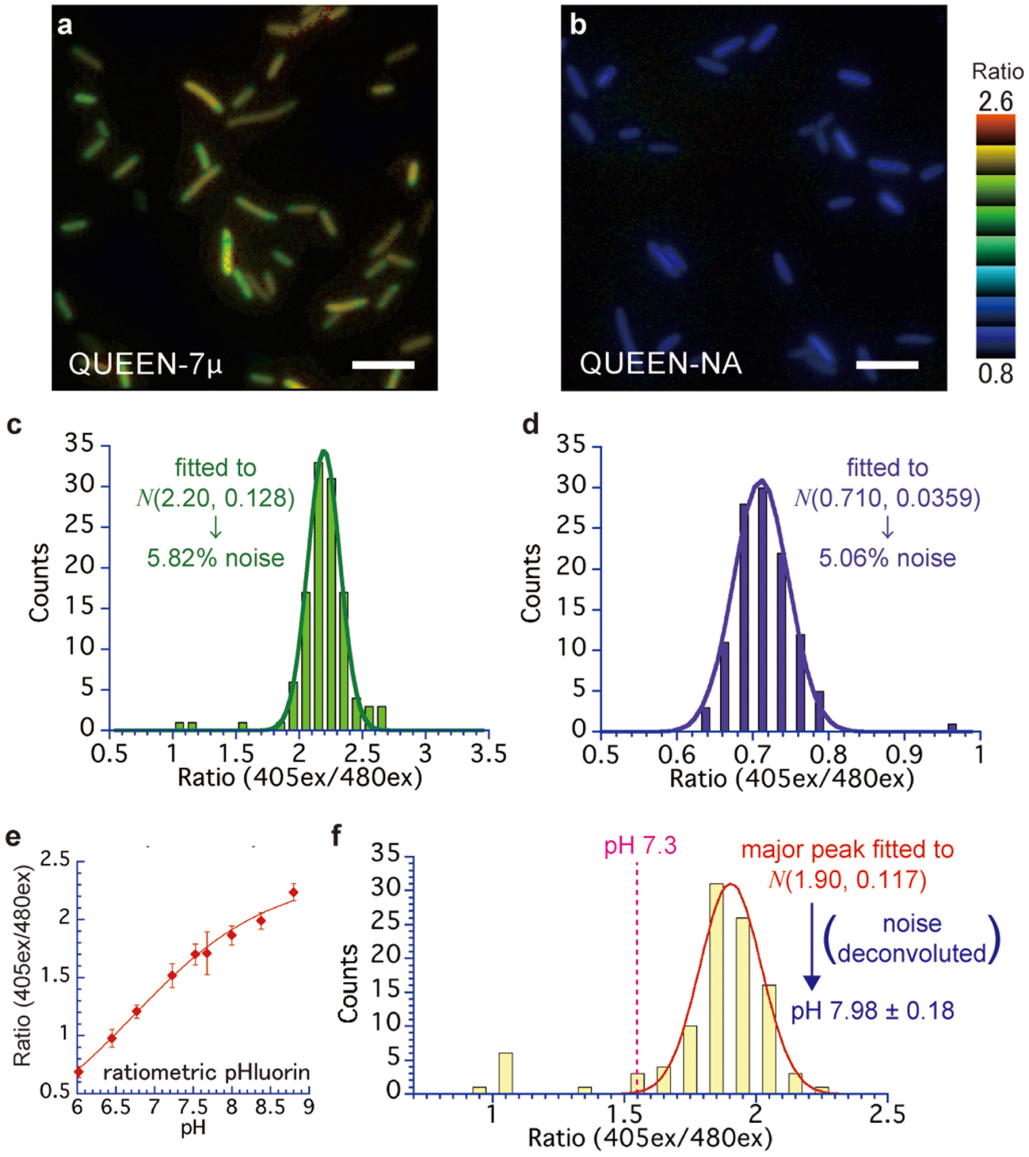
Evaluation of the factors that possibly affect the precision of the measurement by QUEEN. All the cells in this figure are grown in a continuous culture condition. (a–d) The level of noise included in the experimental system. QUEEN variants with constant signal under physiological conditions were used. (a, b) Representative 405ex/480ex ratio images. Bars = 5 µm. (c, d) The distribution of the ratio in each cell population. The distribution was fitted to a normal distribution. The noise level was calculated as SD/mean × 100. (a, c) QUEEN-7µ cells. (b, d) QUEEN-NA cells. (e–f) The pH distribution in the cell population measured by ratiometric pHluorin^18^. (e) The relationship between the ratio and pH value, measured by collapsing the ΔpH between inside and outside of the cell^19^. Error bar = standard deviation (SD). 25–87 cells were examined for each pH value. The data points were fitted to the Hill equation. At pH 8.0, the distribution was fitted to *N*(1.86, 0.089). (f) The distribution of 405ex/480ex ratio in cells expressing ratiometric pHluorin. The main peak was fitted to a normal distribution *N*(1.90, 0.117). In the deconvolution of the noise, the SD of the experimental noise was assumed to be 0.089 (See Supplementary Methods).

As expected, QUEEN-7µ and QUEEN-NA grown in continuous culture showed a nearly uniform 405ex/480ex ratio (Fig. 3a, b, Supplementary Fig. S6). Histograms were made from the data points, and the distributions of ratio were fitted with normal distributions (Fig. 3c, d). The means were 2.20 and 0.71, and the standard deviations were 0.128 and 0.0359, corresponding to 5.82 and 5.06 (%) of the mean for QUEEN-7µ and QUEEN-NA, respectively. Since the standard deviation values normalized by the mean were nearly the same for 2 very different mean ratio values, the level of noise can be assumed to be approximately the same for all ratio values. Therefore, we used the larger value of 5.82 (%) as the noise included in the single-cell QUEEN measurement system. This value was used to correct for experimental noise and obtain true distribution parameters of the ATP distribution in the next section (See Supplementary Methods).

Next, to clarify the effect of diversity in intracellular pH on the QUEEN-2m signal, ratiometric pHluorin^21^ was expressed in *E. coli*. Ratiometric pHluorin is a GFP-based sensor whose 405ex/480ex ratio changes in response to changes in the pH, as confirmed by collapsing the ΔpH and changing the intracellular pH^22^ (Fig. 3e). Cells expressing ratiometric pHluorin were cultured under continuous culture conditions and observed under a microscope (Fig. 3f). One major peak was observed around the ratio 1.9 and one minor peak was observed around the ratio 1. The major peak was fitted by the normal distribution with an average of 1.90 and SD of 0.117, denoted by *N*(1.90, 0.117) hereinafter. When intracellular pH was fixed at 8.0, the distribution of the ratio was *N*(1.86, 0.089) (Fig. 3e). If the distribution observed in this condition is assumed to be the same as the experimental noise in the major peak, the true distribution of the major peak would be estimated to be *N*(1.90, 0.076), which corresponds to a pH value of 7.98 ± 0.18 (mean ± SD) (See Supplementary Methods). Because QUEEN-2m is essentially insensitive to pH changes in the range of 7.3–8.8 (Fig. 1f, g), the pH distribution within the major peak should have little effect on the QUEEN-2m signal. On the other hand, the cells in the minor peak clearly showed low pH, and QUEEN-2m signals in these cells may be biased to higher values. However, only 6.9% (7/102) of the total observed cells showed pH values below 7.3. Accordingly, the effects of these acidified cells on the ATP concentration distribution measurement is expected to be minimal.

### Distribution of ATP concentration in E. coli population

**Figure 4:**
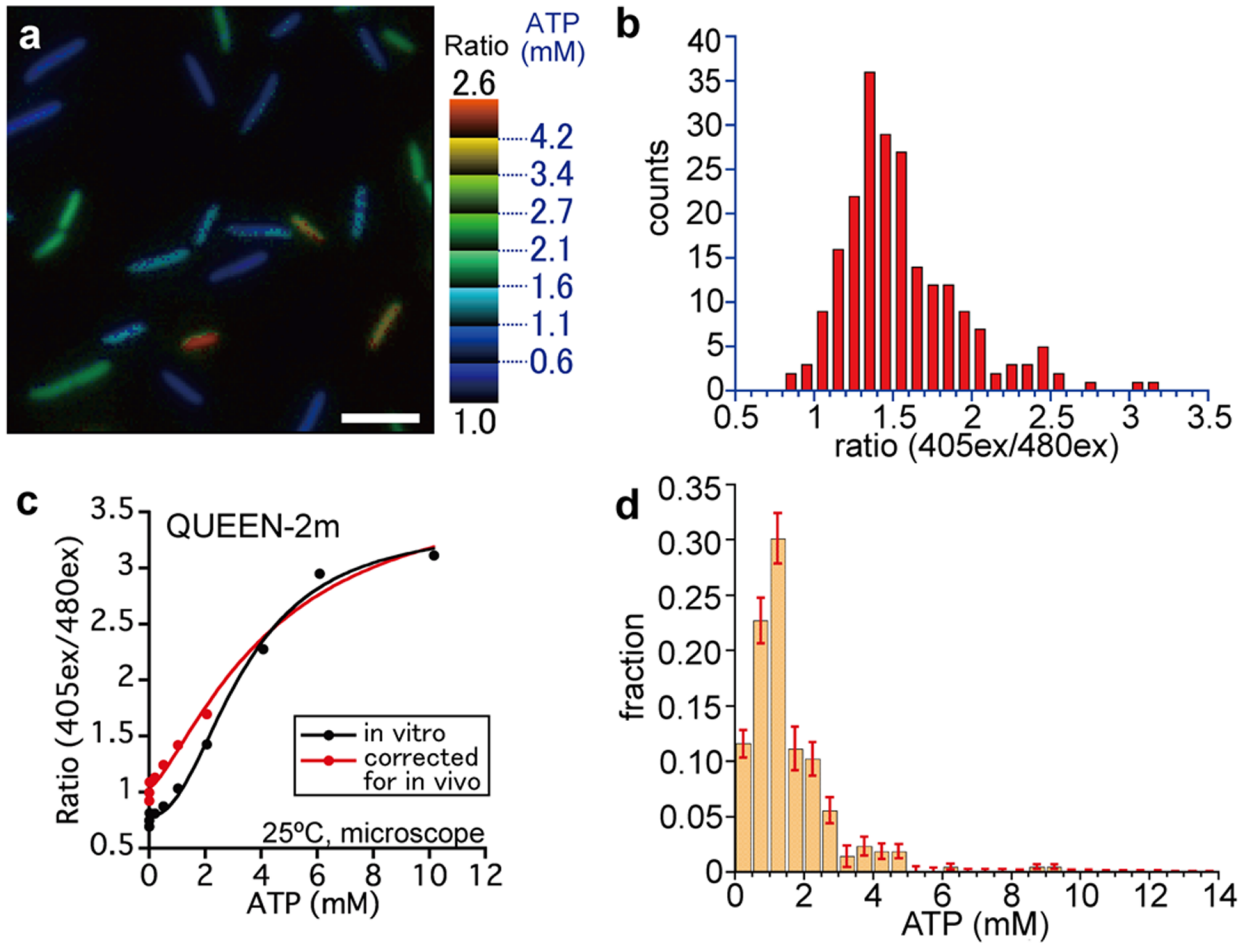
ATP measurement in individual cells by using QUEEN-2m. (a) A representative ratio image of cells expressing QUEEN-2m grown in continuous culture. Bar = 5 µm. (b) Distribution of 405ex/480ex ratio of QUEEN-2m cells in continuous culture in the steady state. (c) The raw (black) and corrected (red) response of 405ex/480ex ratio of purified QUEEN-2m to ATP concentration. Buffer C was used for the measurement. The corrected data show the response of QUEEN-2m in vivo. (d) Distribution of the ATP concentration. Error bars represent the standard deviation (SD) for each column calculated by bootstrapping. Note that 8 out of 216 cells in (b) showed smaller 405ex/480ex value than the ratio value at [ATP] = 0 mM in (c), and thereby assumed to contain 0 mM ATP in (d).

Finally, to determine the absolute ATP concentrations in individual cells, QUEEN-2m-expressing cells were observed under a fluorescent microscope (Fig. 4a). The QUEEN-2m fluorescence signal was not uniform among the cells. Figure 4b shows the histogram of fluorescence signals from single cells, which were cultured in a continuous culture system. The relationship between the 405ex/480ex ratio and the ATP concentration was measured separately using purified QUEEN under the same system, and corrected for in vivo values using the data in Figure 2d (Fig. 4c, Supplementary Methods). Based on this relationship, the fluorescent signals were converted to absolute ATP concentrations (Fig. 4d). The statistics of the distribution were calculated by taking into account the experimental noise obtained in the previous section (Table 1). In this condition, averaged ATP concentration was calculated to be 1.54 mM. Interestingly, it was found that intracellular ATP concentrations were not uniform but significantly diverse between individual cells. Furthermore, the distribution shape of ATP concentrations under this continuous culture condition was not Gaussian, but asymmetric. Skewness is defined as the following for data points *X_1_*, *X_2_*, …, *X_N_*, as a numerical measure of symmetry. 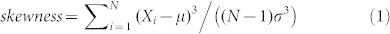
Here, *µ* denotes the average and *σ* denotes the standard deviation. The value of skewness for our measured ATP data was a positive value (2.20) (Table 1), which supports the asymmetric distribution.

## Discussion

FRET-based indicators are widely used to quantify concentrations of substances or enzymatic activities inside living eukaryotic cells. In this study, however, we found that the in vivo signals of a FRET-based ATP indicator, and possibly other FRET-based indicators, could be affected by growth rate, which may be problematic for quantitative imaging of growing bacterial cells. To overcome this problem, we developed a new ratiometric ATP indicator based on single GFP that responds to the absolute ATP concentrations (Fig. 1d). In contrast to FRET-based ATP indicators, our new indicator, QUEEN, is essentially insensitive to cell growth rate (Fig. 2b) and is advantageous for quantification of absolute ATP concentration. Only slight differences were observed between the in vitro and in vivo QUEEN signal (see Fig. 1d, 2b, and 2d), allowing us to obtain an accurate in vivo ATP response curve by applying only a minor correction (Fig. 2d, Supplementary Methods).

Using QUEEN-2m, we successfully determined the ATP distribution in a single population of *E. coli* cells in a continuous culture (Fig. 4d) and calculated statistical parameters (Table 1). The estimated mean and SD values of ATP concentration in our condition was 1.54 ± 1.22 mM (mean ± SD), after correcting for the effect of noise in the experimental system. The data strongly suggested that the individual cells have very different levels of intracellular ATP concentrations. Furthermore, the skewness of the distribution was estimated to be a positive value (2.20), and the ATP concentration showed a long tail towards the high concentration side of the distribution. This suggests that the distribution of ATP concentration is not a symmetric Gaussian-like distribution but rather an asymmetric positively-skewed distribution. Such information on distribution shape has never been reported to our knowledge. Although QUEEN-2m signal may be biased in some cells that are acidified below pH 7.3 (Fig. 3f), the small fraction (6.9%) of such acidified cells in our condition will not largely affect the overall distribution shape of ATP concentrations.

It is important to consider why ATP concentrations are diverse even in a single population of cells that has the same genetic background and is cultured under the same condition. For the number of proteins, it has been proposed that this diversity is derived from the stochasticity of the occurrence of synthesis reactions, i.e., gene expression^2^. In contrast, stochastic fluctuations of ATP synthesis or hydrolysis reactions cannot explain the observed distribution, as these reactions instantaneously reach the steady state (Supplementary Note). It is more likely that the diversity of the enzyme content in each individual cell is the cause for the ATP distribution. Further analysis on the correlation between enzyme number and ATP concentration may elucidate the mechanism resulting in large diversity in ATP concentrations. It should be noted, however, that our results do not exclude the possibility that the different cells have different ATP concentrations but still have similar ATP:ADP ratio. If ATP:ADP ratio is strictly maintained, diversity in ATP concentrations must be the result of diversity in total adenine nucleotide concentrations.

The diversity in the level of ATP may benefit the population by enabling individual cells to adopt different strategies under severe conditions. Some behavior or fate of bacteria cells could be linked with cellular energy levels, and our new ATP indicator may contribute to find them. Future substantiation will involve observing ATP concentration distributions in other conditions and other species to see how it compares with the skewed unimodal distribution of ATP in *E. coli* in our conditions.

## Methods

Details of the gene construction, luciferase assay, conversion of fluorescence ratio to ATP and statistical analysis can be found in Supplementary Methods.

### Chemicals

DNA polymerase was purchased from TaKaRa; restriction enzymes were from Roche; and oligonucleotides, ATP, and ADP were purchased from Sigma-Aldrich. Complementary DNA for ratiometric pHluorin was synthesized by GenScript. All other chemicals were purchased from Wako Pure Chemicals, unless otherwise noted.

### Purification of ATP indicators

*E. coli* strain JM109(DE3) was transformed with the pRSET B vector containing the ATP indicator sequence and cultured in LB medium (100 µg ml^−1^ ampicillin) at 28°C overnight. For QUEEN-2m cells, 1 mM isopropyl β-D-1-thiogalactopyranoside (IPTG) was added to the culture 2 h before harvesting to induce high expression. For the other indicators, IPTG was not added because basal expression was high enough. Harvesting and purification was performed as previously described^13^. The purified proteins were stored at −80°C.

### Characterization of ATP indicators in vitro

The fluorescence of ATP indicators in vitro was tested in buffer C, (50 mM HEPES-KOH [pH 7.7], 200 mM KCl, 1 mM MgCl_2_, and 0.05% Triton X-100), at 25°C unless otherwise stated. Buffer C is designed to be closer to the reported values of pH, K^+^, and free Mg^2+^ found in *E. coli* cytosol^20^,^22^,^23^ compared to buffer B (50 mM MOPS-KOH [pH 7.3], 50 mM KCl, 0.5 mM MgCl_2_, and 0.05% Triton X-100) used in a previous report^13^. A 200 mM stock of ATP-Mg^2+^ or ADP-Mg^2+^ (equimolar mixture of MgCl_2_ and ATP or ADP) was added to the ATP indicator solution to change the ATP-Mg^2+^ concentration, and the fluorescence was measured with a FP8600 spectrophotometer (JASCO), unless otherwise specified. For the spectra measurements, the measurements were started once fluorescence stabilized. When the excitation spectra of single FP-type indicators were measured, the solution was excited at 380–505 nm, and emission was recorded at 513 nm. When the emission spectra of FRET-type indicators were measured, the solution was excited at 435 nm, and emission was recorded at 460–600 nm. For the *k*_on_ and *k*_off_ measurements, single FP-type indicators were excited at 494 nm, and emission at 513 nm was recorded. ATP-Mg^2+^ was added to the solutions during measurement.

### Measurement of ATP indicator in cell suspension

The excitation and emission wavelengths during cell suspension measurement were the same as with the in vitro solution measurements. However, in the cell suspension measurements, fluorescence intensity needs to be corrected for autofluorescence (See Supplementary Methods).

When the fluorescent ATP indicator values in vivo were compared to the in vitro results, the cells grown in batch culture were first diluted to OD_600_ = 0.2. Next, KCN (0, 0.01, 0.05, 0.1, or 10 mM for QUEEN-2m cells; 0, 0.01, 0.1, or 40 mM for ATeam1.03^YEMK^ cells) was added, and the solution was stirred for 10 min. The fluorescence was measured, and finally the sampling for luciferase was performed. The temperature was 25°C for QUEEN-2m and 37°C for ATeam1.03^YEMK^. The effect of high extracellular [ATP] was measured similarly, by adding ATP-Mg^2+^ to the suspension in a stepwise manner. The suspension was stirred for 30 s after each addition of ATP-Mg^2+^, after which the fluorescence was measured.

When the cells in the continuous culture conditions were measured, cells grown in continuous culture at 2 ml of the cell suspension was extracted from the culture and transferred to a cuvette. The fluorescence and OD_600_ values were then measured immediately.

### Microscopic observation

To measure the relationship between the QUEEN 405ex/480ex ratio and ATP concentration under a microscope, purified QUEEN solution in buffer C at various ATP concentration was made to flow between two cover slips separated by greased parafilm separation paper and observed. For the single cell measurement of QUEEN, *E. coli* cells were immobilized on top of a cover slip using a thin agarose film. The film was made by flowing 3% (w/v, in M63 medium) melted agarose into a small space between two cover slips separated by an approximately 0.5-mm-thick polydimethylsiloxane (PDMS) sheet (Supplementary Fig. S5a), and cooling it in a refrigerator. A small amount of cell suspension (3–10 µl) was applied to the cover slip, and the agarose film was placed on top of it. When the film was semi-dry, the cells were immobilized but fluid still bathed the cells (Supplementary Fig. S5b). The advantages of this agarose film are (1) its low toxicity to *E. coli* cells and (2) allowance of gas exchange so that cells can continue aerobic respiration. The immobilized cells were observed with a TE2000 microscope and an ORCA-R2 camera. Epifluorescence imaging was done by combining a xenon lamp, excitation filters (405/20 and 480/40), dichroic mirror (505LP), ×100 objective, and an emission filter (BA510). The exposure time values for 405 nm and 480 nm excitation were 2000 ms and 400 ms, respectively. The temperature was set to 25°C in an INU-NI-F1 incubator (Tokai Hit). The fluorescence image was acquired only once for each cell.

The acquired images were analyzed with Metamorph software (Molecular Devices). Phase-contrast images were taken simultaneously with the epifluorescence. Phase-contrast images were thresholded at the value that was intermediate between the bright background and dark cell and converted to a binary image. The binary image was used as the boundary of the intracellular and extracellular regions in the fluorescence image. The epifluorescence images were processed as follows. First, background intensity was subtracted. Next, the average fluorescence intensity per pixel inside one cell was calculated for each channel. Finally, the ratio was calculated from the average values for each cell. The autofluorescence for each cell measured in pRSET B transformed cells was very low, so the correction of the autofluorescence was not done in the single cell measurements. Instead, QUEEN cells with low fluorescence intensity in either of the two channels were omitted from the data. The details of the conversion of ratio values to ATP concentration are discussed in Supplementary Methods.

In the pH measurement experiments, the microscope setup was the same as above. Ratiometric pHluorin^18^ cells were grown in the continuous culture condition. To construct the reference curve, membrane ΔpH of the cells was collapsed to zero. Cells were resuspended in buffer solution containing M63A medium (0.4 g/l KH_2_PO_4_, 0.4 g/l K_2_HPO_4_, 2 g/l (NH_4_)_2_SO_4_, 7.45 g/l KCl, 2 g/l casamino acids), 40 mM potassium benzoate, 40 mM methylamine hydrochloride, and 100 mM buffer compound^19^. The buffer compound used was MOPS (pH <6.5), HEPES (pH 6.5–8.0), or Tricine (pH >8.0). The 3% agarose membrane to immobilize the cells was made from M63A medium and equilibrated in advance with the buffer solution above. For the actual measurements, membrane ΔpH was not collapsed, and the sampling and observation procedures were the same as with QUEEN cells.

### Cell growth conditions

Unless otherwise indicated, cells in the batch culture condition were cultured in modified M63 medium, (61.5 mM dipotassium hydrogenphosphate, 38.5 mM potassium dihydrogen phosphate, 15.1 mM ammonium sulfate, 1.8 µM iron (II) sulfate, 15 µM thiamine hydrochloride, 0.2 mM magnesium sulfate, 10 mM glucose, 100 µg/ml ampicillin; pH adjusted to 7.0 by potassium hydroxide). In addition, in the QUEEN-2m cell experiments (QUEEN-2m data of Fig. 2d and Supplementary Fig. S3a), 0.04 mM IPTG was included in the medium. The cells were always transformed with the appropriate plasmid 0–3 weeks before starting the culture. The transformed cells were cultured on ampicillin LB plates until colonies formed, stored at 4°C, and inoculated to M63 medium. In the batch culture experiments, unless otherwise noted, cells were repeatedly inoculated to new medium so that the cell OD_600_ was maintained below 0.6 (Growth saturation occurs at OD_600_ 1.1–1.5 under our conditions).

In the continuous culture experiments, the cells were pre-cultured as above in normal M63 medium and then inoculated to low-glucose M63 medium (the same as normal M63, except that the glucose concentration is 1 mM and 0.002% Antifoam 204 [Sigma-Aldrich] is added) in the chemostat. In the QUEEN-2m and ratiometric pHluorin cell experiments, 0.04 mM IPTG was also included in the medium. Hand-made chemostats were constructed from glass media bottles, magnetic stirrers, an isothermal water bath, rubber stoppers, silicone tubes, glass tubes, a peristaltic pump (Atto Corporation), an air pump (AS-ONE), and in-line air filters (SLFG05010, Millipore) (Supplementary Fig. S7). The cultures were constantly bubbled with approximately 500 ml/min of sterile air to provide oxygen. The time of inoculation in the chemostat is defined as time zero. At first, when the OD was very low, medium was not fed to the culture. After the OD exceeded 0.1, medium feeding was started. The chemostat culture volume was maintained within 125–140 ml, and medium feeding was 15 ml/h, giving a dilution rate of 0.10–0.12 h^−1^.

## Author Contributions

H.Y., H.N. and H.I. designed research. H.Y., K.T., K.V.T. and H.I. performed experiments and data analysis. S.K. and T.K. contributed to data analysis. H.Y., S.K., T.K., A.K., H.N. and H.I. prepared the manuscript.

## Supplementary Material

Supplementary Information Supplementary Information

## Figures and Tables

**Table 1:** Statistic parameters of measured ATP concentration distribution. See Figure 4d for the distribution histogram. For each parameter, the upper row (“raw”) is the values directly calculated from the experimental results. The raw data may be biased because of the non-linear property of the ratio-ATP conversion function. The lower row (“corrected”) is the estimated true value with ±1SD estimation error corrected for experimental errors and biases (see Supplementary Methods)

	mean (mM)	SD (mM)	skewness
raw	1.56	1.27	2.63
corrected	1.54 ± 0.09	1.22 ± 0.15	2.20 ± 0.54
