# Supplementary material for: Diversity in ATP concentrations in a single bacterial cell population revealed by quantitative single-cell imaging
Source: Sci Rep. 2014 Oct 6;4:6522. doi: 10.1038/srep06522 (PMC4185378; doi:10.1038/srep06522)
Supplement: Supplementary Information [file srep06522-s1.pdf]

Diversity in ATP concentrations in a single bacterial cell population revealed by quantitative single-cell imaging.

Hideyuki Yaginuma[a,b,c,d], Shinnosuke Kawai[e,f], Kazuhito V. Tabata[a,g], Keisuke Tomiyama[b], Akira Kakizuka[h], Tamiki Komatsuzaki[e], Hiroyuki Noji[a,b,c], Hiromi Imamura[h,i]

([a]Graduate School of Engineering, the University of Tokyo, Japan, [b]Graduate School of Frontier Biosciences, Osaka University, Japan, [c]CREST, JST, Japan, [d]QBiC, RIKEN, Japan, [e]Research Institute for Electronic Science, Hokkaido University, Japan, [f]Faculty of Science, Shizuoka University, [g]PRESTO, JST, Japan, [h]Graduate School of Biostudies, Kyoto University, Japan, [i]The Hakubi Center for Advanced Research, Kyoto University, Japan)

Correspondence: imamura@lif.kyoto-u.ac.jp

Supplementary Information

Supplementary Note

The design of single FP-type ATP indicators

The first single FP-type ATP indicator (QUEEN-7μ) was developed by genetically fusing the ε subunit of the thermophilic *Bacillus* PS3 F_oF_1 ATP synthase to cpEGFP. The amino acid (a.a.) sequence of cpEGFP starts at Y145 and ends at N144, with a 6 a.a. linker (GGTGGS) connecting K238 and M1[18]. The F_oF_1 ε subunit has two α helices in the C-terminal region (a.a. 90–105 and 113–131)[19]. This sequence was split in two, in the middle of the two helices, and the N-terminal side (a.a. 1–107) and the C-terminal side (a.a. 110–133) were fused to the N-terminus and the C-terminus of cpEGFP, respectively (Fig. 1b). Two a.a. linkers were inserted at each junction. The fluorescence properties of the expressed proteins were highly sensitive to the linker a.a. sequences. A random linker library was constructed and screened for indicators with both good fluorescence and a good dynamic range. The variant with the largest

ratio change among the tested candidates was identified and named QUEEN-7μ. This variant had linker sequences of Thr-Arg and Leu-Gly (Fig. 1b).

The ratio of excitation at 400 nm and 494 nm (400ex/494ex ratio) of QUEEN-7μ was responsive to ATP concentration (Fig. 1c, d); however, QUEEN-7μ's affinity for ATP was too high for its use in living bacterial cells.

To reduce the affinity, the ε subunit region of QUEEN-7μ was completely or partially exchanged with the ε subunit of *Bacillus subtilis* F_oF_1, which has a lower affinity for ATP than *Bacillus* PS3 ε[24]. Among the tested variants, one was found to have a good responsiveness to ATP at physiological ATP concentrations (Fig. 1c–e). This variant was named QUEEN-2m and was selected as the main single FP-type indicator. QUEEN-2m has the C-terminal ε sequence a.a. 110–133 from *Bacillus* PS3 and the N-terminal a.a. 1–107 sequence from *B. subtilis* (Fig. 1b). While the shape of the QUEEN-2m excitation spectrum responded to the concentration of ATP (Fig. 1e), the shape of the emission spectrum was almost not affected (Supplementary Fig. S2a). *E. coli* cytosol is reported to have a pH of around 7.7 when the external pH is neutral[22], and the cytosolic Mg^{2+} ion concentration is in the range of 1–2 mM[20]. The QUEEN-2m signal was found to be quite stable under these conditions (Fig. 1f, g, Supplementary Fig. S2b). Analysis of ATP binding kinetics showed that the association and dissociation rate constants of QUEEN-2m at 25°C were k_{on} = 2.7 × 10^{-2} mM^{-1} s^{-1} and k_{off} = 9.4 × 10^{-2} s^{-1}, respectively (Supplementary Fig. S2c).

As a control in later experiments, an ATP-insensitive variant of QUEEN (QUEEN-NA) was also developed. The ε subunit domain of QUEEN-NA was taken from the ε subunit of *B. subtilis* F_oF_1, and it has two mutations in the ATP recognition site of the ε subunit (R122K/R126K) (Fig. 1b). Due to these mutations, the affinity of QUEEN-NA for ATP was completely abolished (Fig. 1c).

The protonated form of the GFP chromophore absorbs ~400 nm light, while the deprotonated form absorbs ~480 nm light. Therefore, it is most likely that ATP binding to the epsilon subunit changes the position of the amino acid residues around

the chromophore in a way that facilitates protonation of the fluorophore. However, we currently do not know the structural details underlying this spectral change. We expect that the precise mechanism will be elucidated by future crystallographic studies.

The effect of ADP to the signals from QUEENs

We measured the effect of ADP to the signal of QUEENs. We noticed that when the concentration of ADP was high, the signals from purified QUEEN-7μ and QUEEN-2m were affected (Supplementary Fig. S1b, e). In case of QUEEN-2m, the signal was biased markedly towards low values in the presence of non-physiologically high (roughly > 1 mM, see the discussion below) concentrations of ADP. In case of QUEEN-7μ, the signal was biased towards low vales at high ATP and ADP levels, but it was biased towards high values at low ATP and high ADP levels. We inferred that the latter apparent increase of QUEEN signal is caused by the contamination of ATP in ADP solution, as ATP and AMP spontaneously forms from ADP.

In order to account for these results, we used the following reaction schemes for QUEEN-7μ.

$$Q\bullet ADP \underset{k_4}{\overset{k_3[ADP]}{\rightleftarrows}} Q \underset{k_2}{\overset{k_1[ATP]}{\rightleftarrows}} Q\bullet ATP \qquad (2)$$

As mentioned above, high concentrations of ADP decreased QUEEN-7μ signal, except for low ATP concentration conditions that the contaminating ATP in ADP solution is non-negligible. Such a result may be explained by assuming a competitive inhibition mechanism of QUEEN by ADP. Thus, in the above scheme, we assumed that ADP can also bind to QUEEN-7μ, but unlike ATP, this does not alter the fluorescence properties of QUEENs. From scheme (2), the following equation can be derived at steady state.

$$\frac{[\mathrm{Q} \bullet \mathrm{ATP}]}{[\mathrm{Q}]_0} = \frac{1}{1 + \left(1 + \frac{[\mathrm{ADP}]}{K_i}\right)\frac{K_d}{[\mathrm{ATP}]}} \quad (3)$$

$[\mathrm{Q}]_0$ is the initial concentration of QUEEN. K_d is the dissociation constant of QUEEN for ATP and equals to $\mathrm{k}_2/\mathrm{k}_1$. K_i is the dissociation constant of QUEEN for ADP and equals to $\mathrm{k}_4/\mathrm{k}_3$. Next, we took into consider the contamination of ATP in ADP solution. Let x and c be the apparent concentration of ADP and ATP added in the solution in our experiments, respectively. The true concentration of ATP is,

$$[\mathrm{ATP}] = \varepsilon x + c \quad (4)$$

where ε is the proportion of ATP contaminated in the ADP solution. The true concentration of ADP is,

$$[\mathrm{ADP}] = (1 - \varepsilon)x \quad (5)$$

In addition, since we assumed that only the ATP-bound QUEEN molecule shows the change in fluorescence ratio,

$$\frac{[\mathrm{Q} \bullet \mathrm{ATP}]}{[\mathrm{Q}]_0} = \frac{\mathrm{R} - \mathrm{R}_{\min}}{\mathrm{R}_{\max} - \mathrm{R}_{\min}} \quad (6)$$

where R is the 400ex/494ex ratio of QUEEN, and $\mathrm{R}_{\min}$ and $\mathrm{R}_{\max}$ is the minimum and maximum values of R, respectively.

Taken together, Eq. (3) can be rewritten as,

$$\frac{\mathrm{R} - \mathrm{R}_{\min}}{\mathrm{R}_{\max} - \mathrm{R}_{\min}} = \frac{1}{1 + \left(1 + \frac{(1-\varepsilon)x}{K_i}\right)\frac{K_d}{\varepsilon x + c}} \quad (7)$$

We used equation (7) to fit the purified QUEEN-7μ data points (Supplementary Fig. S1b). K_d was fixed to 6.86 × 10^{-3} (mM) as obtained by a separate ATP response

measurement (Supplementary Fig. S1a). The fitted values of K_i and ε were 1.24 (mM) and 6.05 × 10^{-3}. From this result, the deviation of 400ex/494ex ratio caused by ADP can be calculated as in Supplementary Figure S1c. When the ATP concentration is high (roughly >1 mM), ADP has almost no effect on 400ex/494ex signal of QUEEN-7μ, so it is safe to use QUEEN-7μ as the constant signal control. For reference, the error level of the single cell measurements under the microscope (5.82%, see Fig. 3 and text) is plotted as the error bars.

Similarly, we considered the following reaction scheme for QUEEN-2m.

$$\mathrm{Q{\bullet}ADP} \underset{k_4}{\overset{k_3[\mathrm{ADP}]}{\leftrightarrows}} \mathrm{Q} \underset{k_2}{\overset{k_1[\mathrm{ATP}]^2}{\rightleftarrows}} \mathrm{Q{\bullet}ATP_2} \qquad (8)$$

Note that for QUEEN-2m, we assumed that the cooperativity of ATP binding is 2, since the fitting of the response of QUEEN-2m signal to ATP by a Hill equation gives a Hill coefficient value close to 2 (Fig. 1d). In addition, the slight contamination of ATP in ADP should have very little effect on QUEEN-2m signal, so it was not considered here. Then, the following equation can be derived from this scheme:

$$\frac{\mathrm{R}-\mathrm{R}_{\min}}{\mathrm{R}_{\max}-\mathrm{R}_{\min}} = \frac{1}{1+\left(1+\frac{[\mathrm{ADP}]}{K_i}\right)\left(\frac{K_d}{[\mathrm{ATP}]}\right)^2} \qquad (9)$$

We have also considered a scheme that assumes the cooperativity of ADP binding to be 2, but the equation derived from it did not fit to the experimental data well.

Equation (9) was used to fit the purified QUEEN-2m data points (Supplementary Figure S1e). K_d was fixed to 2.44 (mM) as obtained by a separate measurement (Supplementary Fig. S1d). The fitted value of K_i was 3.17 (mM). From this result, the deviation of 400ex/494ex ratio caused by ADP was calculated for several true ATP concentrations (Supplementary Fig. S1f). In addition, the deviation

of apparent [ATP] value was calculated as in Supplementary Figure S1g. Again, the error of 5.82% in the 400ex/494ex ratio value was plotted as the error bars for reference.

Previously, Tran and Unden measured the amount of ATP and ADP inside *E. coli* cells under various culture conditions[25]. According to their data, the average ADP inside *E. coli* is always in the range of 0 to 2.0 μmol/gDW, where gDW stands for gram dry weight. This held true regardless of whether aerobic or anaerobic culture conditions, or whether the cells were in rapid growth (log phase) or slow growth (stationary phase) conditions. Assuming intracellular volume of 0.0023 l/gDW[26], 2.0 μmol/gDW corresponds to 0.87 mM. We can see from Supplementary Figure S1g that this level of deviation is trivial for the low or high ATP concentration condition, and is within the range of experimental errors. The effect of ADP may be non-trivial for the intermediate levels of ATP concentrations (roughly 1-5 mM) where the 400ex/494ex ratio responds sharply to the change of ATP, but the bias is at a maximum ~10% for 0.87 mM of ADP. Of course, the previously measured ADP values are the average values of a number of cells, and there is the possibility that minor population of individual bacteria cells have much higher ADP concentrations. However, this minor population would have only a small effect on ATP distribution. Therefore, we concluded that even ADP effect was taken into consider, our point that each cells have different ATP levels still holds. We do not think that the asymmetric ATP distribution shown in Fig. 4d is explainable only by the ADP effect either.

The effects of growth rate change and maturation/degradation kinetics to the signals of ATeam

We have shown that the ratio of emission at 527 nm and 475 nm (527em/475em) of ATeam is lower in vivo than in vitro (Fig. 2d). We consider this to be an intrinsic problem of a FRET-based indicator composed of two fluorescent proteins. Here, we will discuss the mechanism of this signal bias in detail and how it can explain the signal changes of AT3.10 seen in Figure 2b. As discussed in the main

text, the low in vivo ratio may be explained by the existence of half-mature or half-degraded FRET-based indicators or both[27].

First, because FPs can take several hours after translation to mature and become fluorescent, half-mature biosensors with only one of the two FPs showing fluorescence inevitably exist in the population. The time required for maturation depends on the types of FPs, with yellow FPs (YFPs) tending to require a longer time to mature compared to cyan FPs (CFPs)[28,29]. Therefore, it is expected that a substantial proportion of the ATeam molecules have immature YFPs and behave like a single CFP. Obviously, FRET does not occur in these half-mature ATeams. The total YFP/CFP ratio of the sample is low when these half-mature ATeams are present. This is not a problem with the in vitro measurements, as the half-mature ATeams will fully mature during the purification process.

Second, intracellular proteolytic systems can cleave FRET-based biosensors, yielding half-degraded biosensors and abolishing FRET between the CFP and the YFP. If these half-degraded FPs remain fluorescent, then the YFP/CFP ratio will be reduced.

If these half-mature and/or half-degraded ATeams actually exist, the fluorescence signals of the FRET-type ATeam in vivo will be affected by the growth rate change. This hypothesis may be investigated by the following simple mathematical model and simulation described below. Consider the following kinetic scheme:

$$\varnothing \xrightarrow{a\mu} \underset{\text{(half-mature)}}{\mathrm{X}} \xrightarrow{k_1} \underset{\text{(fully-mature)}}{\mathrm{Y}} \xrightarrow{k_2} \underset{\text{(half-degraded)}}{\mathrm{Z}} \xrightarrow{k_3} \varnothing \qquad (10)$$

Here, X, Y, and Z represent the different states of ATeams. X is the half-mature ATeam with fluorescent CFP but with non-fluorescent YFP. Y is the fully mature ATeam. Z is the half-degraded but still fluorescent ATeam. The empty set symbol ($\varnothing$) denotes the non-existence of the ATeam. We assumed that the emergence rate of half-mature ATeam is proportional to cell growth rate μ by

proportional constant a. k_1 is the maturation rate of half-mature ATeam to fully-mature ATeam, k_2 is the degradation rate of fully-mature ATeam to half-degraded but fluorescent ATeam, and k_3 is the degradation rate of half-degraded ATeam to non-fluorescent fragments.

On the basis of the above scheme, we attempt to explain the signal changes observed in the continuous culture of AT3.10 (Fig. 2b) below. In the case of a batch culture where there is no medium flow into or out of the reactor, the total cell volume in the reactor increases by a rate proportional to the current total cell volume (x) and the growth rate (μ), as long as the extracellular glucose concentration is sufficient.

$$\frac{dx}{dt} = \mu x \qquad (11)$$

In contrast, in the continuous culture system, medium flows into and out of the reactor at a constant speed. Therefore, x increases due to cell growth, but decreases due to removal of cell suspension to waste, expressed in the following differential equation as:

$$\frac{dx}{dt} = \mu x - \frac{F}{V} x \qquad (12)$$

where F is the speed of medium flowing out of the reactor and V is the volume of the reactor. Since V is constant in the continuous culture, the rates of media flowing in and out of the reactor, F_{in} and F_{out}, are equal ($F = F_{in} = F_{out}$). The ratio of medium flow to reactor volume (F/V) is called the dilution rate. From the above equation, at a steady state, the growth rate is equal to the dilution rate.

$$\mu = \frac{F}{V} \qquad (13)$$

The total amount of X in the continuous culture system follows the differential equation below:

$$\frac{d([\mathrm{X}]x)}{dt} = a\mu x - k_1[\mathrm{X}]x - [\mathrm{X}]x\frac{F}{V} \tag{14}$$

The first term on the right side describes the production of X. The second term describes the disappearance of X by maturation. The last term describes the outflow of cells containing X from the reactor. At a steady state, Eq. (14) can be simplified using Eq. (13) to

$$\frac{d([\mathrm{X}])}{dt} = a\mu - k_1[\mathrm{X}] - \mu[\mathrm{X}] \tag{15}$$

Similarly,

$$\frac{d([\mathrm{Y}])}{dt} = k_1[\mathrm{X}] - k_2[\mathrm{Y}] - \mu[\mathrm{Y}] \tag{16}$$

$$\frac{d([\mathrm{Z}])}{dt} = k_2[\mathrm{Y}] - k_3[\mathrm{Z}] - \mu[\mathrm{Z}] \tag{17}$$

Dilution rate can be easily controlled in the continuous culture system. Suppose the system is at a steady state at a certain dilution rate ($F/V = \mu = \mu_1$) and the extracellular glucose concentration is very low. By setting the dilution rate to a smaller value, the growth rate can be slowed. If we suddenly change μ to a new value ($\mu = \mu_2 < \mu_1$) we expect [X], [Y], and [Z] to change until they reach new steady state values, and as a result, we should see a change in the total ATeam signal as well.

To predict changes in the proportions of the different ATeam states during the continuous culture, we simulated the kinetic scheme, setting the parameters to biologically relevant values. In the simulation, μ values were changed from $\mu_1 = 0.25$ to $\mu_2 = 0.1$ at $t = 5$ h. k_2 and k_3 values were set to values smaller than k_1, because the degradation of ATeam is slow, (otherwise, the yield of ATeam purification from *E. coli* culture would be extremely low). In this simulation, we observed a decrease in [X] and an increase in [Y], followed by a gradual decrease in [Y] and a gradual increase in [Z]

(Supplementary Fig. S3a). One type of ATeam, AT3.10, had a saturated ATP-binding site at physiological ATP concetrations (Fig. 2a). Thus, the signal of AT3.10 would not be affected by ATP concentrations when expressed in cells. We assumed that the relative fluorescence intensities expected to linearly correlate with the concentration of each AT3.10 state in CFP and YFP channels were 200 (for X_{CFP}), 70 (X_{YFP}), 95 (Y_{CFP}), 220 (Y_{YFP}), 200 (Z_{CFP}), 170 (Z_{YFP}). These values are approximately proportional to the measured in vitro fluorescence intensities of AT3.10 and singular FPs. Note that emission from singular CFPs partially flows into the YFP channel, and singular YFP could be weakly excited by the CFP excitation light. Using these values, we simulated the YFP/CFP ratio change of AT3.10 in the continuous culture system (Supplementary Fig. S3b). According to the simulation results, after the growth rate slowdown, the YFP/CFP ratio first increased and then gradually decreased. The first rise in the ratio corresponds to a decrease in half-mature ATeam, while the following gradual decrease corresponds to an increase in half-degraded ATeam.

The experimental data for AT3.10 (Fig. 2b) could be explained well by this scheme. Initially, when the cell density is low and cells can divide—as in the log phase of the batch culture—μ is approximately 0.25 (according to a separate batch culture measurement). When the OD approaches 0.2, growth gradually becomes limited by the available glucose in the medium flowing into the reactor. Eventually, the system reaches a steady state, and at this phase, the growth rate slows to μ = 0.10–0.12 (= dilution rate of the culture). After this slowdown, we observed a gradual decrease in the AT3.10 signal (Fig. 2b). This corresponds to the gradual ratio decrease in the simulation (Supplementary Fig. S3b). In addition, the AT3.10 signal of the cells in the log phase was measured and found to be 1.99 ± 0.05 in a separate batch culture measurement, which is smaller than the signal after the growth rate slowdown, (2.27 ± 0.04 at t = 25 in Fig. 2b). This suggests that the AT3.10 signal shows a transient increase, again like the simulation. Thus, we concluded that both half-mature and half-degraded ATeam did indeed affect the ATeam signal.

Interpretations for the insensitivity of QUEEN to growth rate change

In contrast to ATeam, QUEEN-7μ expressed in cells had almost the same ratio value throughout the continuous culture (Fig. 2b). According to the scheme described above, this suggests that neither half-mature nor half-degraded QUEEN has any substantial effects on the total QUEEN signal. It is obvious why half-mature QUEEN does not affect the signal, but why does half-degraded QUEEN not have any significant effect on the total signal? This asymmetrical effect of half-degraded sensors may stem from the difference in intrinsic disorder in the two proteins. It is believed that intrinsically disordered regions (IDRs), which natively have no rigid structure, are especially sensitive to degradation[30]. In the ATeam molecule, there are at least two potential IDRs. The first is the "hinge" between the two C-terminal α helices of the ε subunit (a.a. 103–112 in ε numbering), which shows quite a disordered structure when ATP is not bound[19]. This hinge is partially substituted by cpEGFP in QUEEN (Fig. 1a, b). Therefore, the hinge region may be masked from protease attack by cpEGFP. In addition, it is also likely that the new hinge of QUEEN is less flexible and thus less susceptible to proteolysis, because in order to transduce the conformational change of the ε subunit to cpEGFP, the linker region between them should be relatively rigid. It is likely that a linker sequence with less disorder was selected in the screening process. The second potential IDR is in the region of the circularly permuted FP (cpFP), which connects the original C- and N- termini ("Ct-L-Nt" region). In the cpEGFP crystal structure, 19 a.a. in this region are missing[31] and are therefore predicted to be disordered. Because the β-barrel structures of cpFPs are very rigid, cpFPs may retain their fluorescence even after they are cleaved in the Ct-L-Nt region. Even if cpFPs lose their fluorescence by this cleavage, the total QUEEN signal will not be affected, as the cleaved QUEEN will no longer be fluorescent. In contrast, if an ATeam molecule is cleaved at the Ct-L-Nt region of the acceptor cp173-mVenus (a kind of circularly permuted YFP), and this results in the loss of acceptor fluorescence, the half-degraded ATeam would behave like a single CFP and affect the total ATeam signal. Taking these together, we believe that the single FP-based QUEEN is not only resistant to half-mature sensor effects, but is also mostly insensitive to half-degraded sensor effects compared to FRET-type ATeam.

The dynamic range of QUEEN-2m in vivo

In Figure 2d, we could not obtain data points for ATP concentration greater than 5 mM, and the observed maximum ratio of QUEEN-2m 400ex/494ex was approximately 3. However, by adding a high concentration of ATP to the extracellular solution, we can expect that at least some of this ATP is taken up into the cell. We observed that in vivo, the QUEEN-2m ratio signal rose to ≃4.0 when up to 10 mM ATP was added to the cell suspension (Supplementary Fig. S4a), even though the precise intracellular ATP concentration in this condition is unknown. The minimum 400ex/494ex ratio in vivo was 1.3, from Fig. 1d. Thus, the dynamic range of QUEEN-2m in vivo is at least 3.0-fold.

In contrast, when a high concentration of ATP was added to the extracellular solution of AT1.03^{YEMK}, the in vivo 475em/527em signal did not substantially increase (Supplementary Fig. S4b). Therefore, we calculated the minimum and maximum signals of AT1.03^{YEMK} to be 0.99 and 1.8, respectively, and the dynamic range to be 1.8-fold. The reason for the slight decrease in the signal at extracellular ATP concentration higher than 5 mM is unknown.

Timescale of ATP synthesis and hydrolysis rate fluctuations

In the case of protein concentration, the main factors underlying its distribution are the stochastic fluctuations in the processes of mRNA transcription and protein translation[2]. Is it possible to explain the [ATP] distribution from the stochastic fluctuations of ATP synthesis and hydrolysis processes in a similar way? Considering that the timescales of ATP synthesis and hydrolysis are much faster than those of protein synthesis and degradation, this does not seem to be the case.

From one gene, mRNAs are transcribed 0.1–10 times per cell cycle[2]. They are degraded immediately (τ = 1.5 min) and never accumulate within the cell[32]. In contrast, once proteins are translated from mRNAs, the speed of protein degradation is usually slow and negligible, compared to the rate of dilution due to growth[2]. In our

growth conditions (25°C, M63 minimum medium), the doubling time at the log phase was approximately 2.8 h. Taking into account all these factors, the cells are estimated to transcribe 0.036–3.6 mRNAs per hour, and the protein dilution rate is estimated to be 0.78 h^{-1}. As a consequence, the protein concentration fluctuates over the timescale of several tens of minutes to several hours.

In contrast, the rate of ATP turnover inside bacteria is very fast. ATP consumption rate (r_{ATP}) can be calculated from the following linear function,

$$r_{ATP} = \mu / Y + m \tag{18}$$

Here, Y is the yield of cell mass per mole of ATP, and m is the amount of ATP required for maintenance of the cell per unit time and unit mass[33,34]. According to Hempfling and Mainzer, Y = 10.3 gDW (mol ATP)$^{-1}$ and m = 18.9 mmol ATP gDW^{-1} h^{-1} for *E. coli* cells grown in glucose-containing medium[35]. If we accept a volume per unit mass of 2.3 ml gDW^{-1} for *E. coli* cells[26] and assume that the relative growth rate (μ) is 0.10 h^{-1}, we obtain r_{ATP} = 3.5 mM ATP s^{-1}. In our measurements, the average intracellular ATP concentration in log-phase cells was 1.5 ± 0.1 mM. The characteristic time of this system is probably less than 1 s, which means that any stochastic perturbation to the system would be completely relaxed within seconds. Therefore, we conjecture that the stochastic fluctuations of synthesis or hydrolysis reactions have negligible effects on the ATP concentration distribution of the bacterial population. Instead, the difference in ATP synthesis or hydrolysis activities might be the main cause of ATP concentration distribution between cells.

Supplementary Methods

Gene construction and screening

To construct the random linker library for the single FP-type ATP indicator, the N-terminal region (a.a. 1–107) of the *Bacillus* PS3 F_oF_1 ε subunit sequence was amplified from the AT3.10 plasmid[13] by PCR, using forward and reverse primers that have a 15–20-b overlapping sequence with the pRSET B vector (Invitrogen) and cpEGFP, respectively (primer#1 and #2, see the list below for oligonucleotide sequences). The reverse primer contained a 6-b (2 a.a.) random sequence linker between the cpEGFP and the ε subunit sequences. The C-terminal region (a.a. 110–133) of the ε subunit was similarly amplified using a forward primer with a cpEGFP sequence and 6-b random sequence, and a reverse primer with a pRSET B sequence (primer#3 and #4). The cpEGFP sequence was also amplified by PCR using primer#5 and #6. The amplified DNAs were then fused as in Figure 1b, by overlap PCR, and the product was cloned into pRSET B vector using *Bam*HI and *Eco*RI.

Next, the random linker library was screened for single FP-type indicators that showed a large dynamic range. *E. coli* strain JM109(DE3) (Promega) was transformed with the library, and colonies with visible green fluorescence under blue or violet light were selected visually. The His-tagged single FP-type indicator candidates were then crudely purified from the selected colonies using the MagneHis Protein Purification System (Promega), and the excitation spectra in the absence and presence of ATP (0.01, 0.1, or 1 mM) were recorded. The candidate that had the largest dynamic range was termed QUEEN-7μ.

We next constructed a variation of the single FP-type indicator, termed QUEEN-sp1.03. Both N-terminal and C-terminal regions of QUEEN-sp1.03 have the sequence originally from the *Bacillus subtilis* F_oF_1 ε subunit, which shows lower affinity for ATP. First, to construct a vector containing only the *B. subtilis* F_oF_1 ε subunit, the *B. subtilis* ε sequence was amplified using the AT1.03 plasmid[13] as the template. The forward primer (primer#7) was designed to have 15-b extensions homologous to the pRSET B vector *Bam*HI site and the reverse primer (primer#8) to be homologous to the *Hind*III site. The pRSET B sequence was also amplified with the forward primer at the *Hind*III site and reverse primer at the *Bam*HI site (primer#9 and #10). Subsequently, the amplified vector and *B. subtilis* ε were joined using the in-Fusion HD Cloning Kit (Clontech). This pRSET B-*B. subtilis* ε plasmid was amplified using forward and reverse primers homologous to a.a. positions 110 and 107 of *B. subtilis* ε, respectively (primer#11 and #12). The cpEGFP sequence, including the 2 a.a. linkers, was also amplified by PCR using primers with extension sequences homologous to *B. subtilis* ε (primer#13 and #14). By joining the PCR products with in-Fusion, we obtained pRSET B-QUEEN-sp1.03. Unfortunately, the affinity of QUEEN-sp1.03 for ATP was too weak, and QUEEN-sp1.03 could not measure mM levels of ATP.

We constructed QUEEN-2m as follows. The sequence, including the N-terminal region of *B. subtilis* ε and cpEGFP with linkers, was amplified using pRSET B-QUEEN-sp1.03 as a template. The forward and reverse primers had 15-b. extension sequences homologous to the *Bam*HI site of the pRSET B vector and *B.* PS3 ε, respectively (primer#7 and #15). The sequence that includes the C-terminal region of *B.* PS3 ε and most of the pRSET B vector was also amplified using the pRSET B-QUEEN-7μ plasmid as a template. The forward and reverse primers were homologous to a.a. position 110 of *B.* PS3 ε and BamHI site of pRSET B, respectively (primer#16 and #10). The PCR products were then connected by in-Fusion again to obtain pRSET B-QUEEN-2m.

QUEEN-NA, an ATP-insensitive variant of QUEEN, was constructed by a similar method as QUEEN-sp1.03. The only difference was the PCR template for the amplification of the ε sequence; this time, the AT1.03RK plasmid[13] was used as the template.

The list of oligonucleotides used in the above steps is provided below. The final amino acid sequences of the QUEENs used in this manuscript are also shown for convenience.

Primer No.	Sequence (5' to 3')
#1	ATAAGGATCCGATGAAAACGATCCACGTGAGC
#2	GTGGCTGTTGTANNNNNNCTGGCTTTGCAGGCGGCGCTC
#3	AAGCTGGAGTACAACNNNNNNGACATCGACTTCAAACGG
#4	TTCGAATTCACTTCATTTCCGCAACGCTC
#5	CTGCAAAGCCAGNNNNNNTACAACAGCCACAACGTCTAT
#6	GAAGTCGATGTCNNNNNNGTTGTACTCCAGCTTGTGCCC
#7	ACGATAAGGATCCGATGAAAACTGTGAAAG
#8	AGCCGGATCAAGCTTTCAGTTTGCCTTCCCAGCCAC
#9	AAGCTTGATCCGGCT
#10	CGGATCCTTATCGTC
#11	GACACCGATATTCGCCGG
#12	TTGAGAGTTCAGCCGCTC
#13	CGGCTGAACTCTCAAACCCGTTACAACAGCCAC
#14	GCGAATATCGGTGTCACCCAGGTTGTACTCCAG
#15	TTTGAAGTCGATGTCACCCAGGTTGTACTCCAG
#16	GACATCGACTTCAAACGG

QUEEN-7micro a.a. sequence
MKTIHVSVTTPDGPVYEDDVEMVSVKAKSGELGILPGHIPLKAPLEISAARLKKGGKTQYIAVSG
GNLEVRPDKVTINAQAAERAEDIDVLRAKAAKERAERRLQSQTRYNSHNVYIMADKQKNGIKVN
FKIRHNIEDGSVQLADHYQQNTPIGDGPVLLPDNHYLSTQSKLSKDPNEKRDHMVLLEFVTAAGI
TLGMDELYKGGTGGSMVSKGEELFTGVVPILVELDGDVNGHKFSVSGEGEGDATYGKLTLKFIC
TTGKLPVPWPTLVTTLTYGVQCFSRYPDHMKQHDFFKSAMPEGYVQERTIFFKDDGNYKTRAEV
KFEGDTLVNRIELKGIDFKEDGNILGHKLEYNLGDIDFKRAELALKRAMNRLSVAEMK*

QUEEN-2m a.a. sequence
MKTVKVNITTPDGPVYDADIEMVSVRAESGDLGILPGHIPTKAPLKIGAVRLKKDGQTEMVAVSG
GTVEVRPDHVTINAQAAETAEGIDKERAEAARQRAQERLNSQTRYNSHNVYIMADKQKNGIKVN
FKIRHNIEDGSVQLADHYQQNTPIGDGPVLLPDNHYLSTQSKLSKDPNEKRDHMVLLEFVTAAGI
TLGMDELYKGGTGGSMVSKGEELFTGVVPILVELDGDVNGHKFSVSGEGEGDATYGKLTLKFIC
TTGKLPVPWPTLVTTLTYGVQCFSRYPDHMKQHDFFKSAMPEGYVQERTIFFKDDGNYKTRAEV
KFEGDTLVNRIELKGIDFKEDGNILGHKLEYNLGDIDFKRAELALKRAMNRLSVAEMK*

QUEEN-NA a.a. sequence
MKTVKVNITTPDGPVYDADIEMVSVRAESGDLGILPGHIPTKAPLKIGAVRLKKDGQTEMVAVSG
GTVEVRPDHVTINAQAAETAEGIDKERAEAARQRAQERLNSQTRYNSHNVYIMADKQKNGIKVN
FKIRHNIEDGSVQLADHYQQNTPIGDGPVLLPDNHYLSTQSKLSKDPNEKRDHMVLLEFVTAAGI
TLGMDELYKGGTGGSMVSKGEELFTGVVPILVELDGDVNGHKFSVSGEGEGDATYGKLTLKFIC
TTGKLPVPWPTLVTTLTYGVQCFSRYPDHMKQHDFFKSAMPEGYVQERTIFFKDDGNYKTRAEV
KFEGDTLVNRIELKGIDFKEDGNILGHKLEYNLGDTDIRRAELALQKALNKLDVAGKAN*

Correction for autofluorescence in cell suspension measuements

The intensity of *E. coli* autofluorescence was measured using the cells transformed with the empty pRSET B vector or pRSET B-*B. subtilis* ε vector cultured in the same conditions. Both types of non-fluorescent cells showed similar results. The autofluorescence at each excitation/emission wavelength was linearly correlated with the OD_{600} of the cell suspension in our conditions. Therefore, the autofluorescence intensity was estimated from OD_{600} and subtracted from the fluorescence intensity of

cells expressing ATeam or QUEEN. The corrected intensity of each channel was then used to calculate the ratios 400ex/494ex or 527em/475em. The standard error in the estimates were also calculated and are shown in Figure 2b and 2d as vertical error bars (sometimes error bars are small and are hidden by the symbols). The details of the calculation are explained below.

If we assume that the autofluorescence of the ith non-fluorescent cell sample in channel k (k: 400ex, 494ex, 527em, or 475em) is linearly correlated to the non-fluorescent cell OD (p_i), the autofluorescence ($q_{i,k}$) can be written as,

$$q_{i,k} = a_k + b_k p_i + e_{i,k} \tag{19}$$

Here, a_k and b_k are constant values, and $e_{i,k}$ is the residual error of fitting. By fitting the empty vector cell data (total m samples) using the least squares method, a_k and b_k were determined. The standard deviation of the residual error (standard error of regression) in autofluorescence estimation ($s_{q,k}$) is calculated as,

$$s_{q,k} = \sqrt{\frac{\sum_{i=1}^{m} {e_{i,k}}^2}{m-2}} \tag{20}$$

The intensity of the jth fluorescent ATP indicator cell sample of channel k ($y_{j,k}$) can be considered as the sum of the estimated autofluorescence intensity ($\hat{q}_{j,k}$) and the estimated true ATP indicator intensity ($\hat{y}_{j,k}$).

$$y_{j,k} = \hat{y}_{j,k} + \hat{q}_{j,k} \tag{21}$$

From the empty vector cell results, when the OD of the jth sample is x_j,

$$\hat{q}_{j,k} = a_k + b_k x_j \tag{22}$$

Therefore,

$$\hat{y}_{j,k} = y_{j,k} - a_k - b_k x_j \tag{23}$$

In the comparison experiment of ATP indicator and luciferase assay results, x_j was fixed at 0.2, whereas in the time course measurement of chemostat, x_j varied.

We did not consider the error in $y_{j,k}$; repeating the measurement of the same sample at the same time point gave a very small deviation. Thus, the standard error of the estimated true intensity ($s_{\hat{y},j,k}$) is the same as the standard error of the autofluorescence estimation.

$$s_{\hat{y},j,k} = s_{q,k} \tag{24}$$

The fluorescence intensity ratio of two channels (α,β) for sample j ($\hat{Z}_{j,\alpha,\beta}$) was calculated as,

$$\hat{Z}_{j,\alpha,\beta} = \frac{\hat{y}_{j,\alpha}}{\hat{y}_{j,\beta}} \tag{25}$$

By the error propagation law, the standard error in the ratio ($s_{\hat{Z},j,\alpha,\beta}$) was calculated as,

$$s_{\hat{Z},j,\alpha,\beta} = \hat{Z}_{j,\alpha,\beta}\sqrt{\left(\frac{s_{\hat{y},j,\alpha}}{\hat{y}_{j,\alpha}}\right)^2 + \left(\frac{s_{\hat{y},j,\beta}}{\hat{y}_{j,\beta}}\right)^2} = \hat{Z}_{j,\alpha,\beta}\sqrt{\left(\frac{s_{q,\alpha}}{\hat{y}_{j,\alpha}}\right)^2 + \left(\frac{s_{q,\beta}}{\hat{y}_{j,\beta}}\right)^2} \qquad (26)$$

Luciferase assay

For the luciferase assay, 20 µl of cell suspension was sampled from the culture and immediately mixed with 180 µl of luciferase sampling buffer (100 mM Tris-HCl, pH 7.75; 4 mM ethylenediaminetetraacetic acid) preheated to 95°C. The sampling was repeated 3–5 times for the same cell suspension sample. The mixture was incubated at 95°C for 3 min, transferred to ice, and then frozen with liquid nitrogen. The sample was stored at -80°C until assay. The luciferase assay was performed using the ATP Bioluminescence Assay Kit CLS II (Roche) according to the manufacturer's instructions. The measurement of bioluminescence was done using FlexStation FS3 (Molecular Devices). The average (l_{cs}) and SD (s_{cs}) of the cell suspension sample were calculated. To measure [ATP] outside of the cells, the cell suspension was centrifuged, and the supernatant was also assayed in the same way. The average (l_{sup}) and SD (s_{sup}) of the supernatant were also calculated. To correct for the effects of cell components on the reaction, a known amount of ATP was added to the mixture of sampling buffer and cell suspension and measured by luciferase assay. By averaging 7 such measurements, the correction factor (γ) was calculated. The SD was regarded as the error of the correction factor (s_g).

To calculate the total cytosol volume in the suspension, the number of cells and the average volume of the cells were determined simultaneously, with luciferase sampling. The number of cells per unit volume ($\overline{N}$) was counted using an SLGC hemocytometer (Sunlead Glass), 0.4–0.6 mm thick cover glass (Matsunami), and an IX81 microscope (Olympus) equipped with a ×20 objective. The counting was performed on the same sample at 3 different time points, and the SD was considered as the error ($s_{\overline{N}}$). For the determination of the average cell volume ($\overline{V}$), the cell membranes were stained with fluorescent ATTO 655 dye. Cells were collected by centrifugation and washed with and suspended in 0.1 M sodium hydrogen carbonate. To this, 1 µM ATTO 655 NHS ester (Sigma-Aldrich) dissolved in DMSO was added, and the solution was incubated for 10 min at room temperature, washed 3 times with M63 medium, and observed under a TE2000 epifluorescence microscope (Nikon) equipped with a ×100 objective. The optical parts used were a 615/45 excitation filter, FF660-Di01 dichroic mirror (Semrock), and 692/40 emission filter. Images were acquired with an ORCA-R2 camera (Hamamatsu). With this procedure, the membrane could be visualized as a fluorescent layer, and we assumed that the peak of the fluorescence in the layer was the exact position of the membrane (Supplementary Fig. S8a, b). The short axis and long axis of the cytosol of each cell were determined from the image, and the cell volume was calculated by assuming that the cells were cylindrical (Supplementary Fig. S8c). The average of 3 different positions in each cell were used as the short axis. The long axis was measured only once per cell because the effect of measurement error on the volume is small compared to that of the short axis. More than 60 cells were analyzed in this way, and their standard error of the mean was regarded as the error, ($s_{\overline{V}}$).

The concentration of the ATP inside cytosol (A_{luc}) and its standard error (s_{luc}) were calculated as,

$$A_{luc} = \frac{10\gamma\left(l_{cs} - l_{sup}\right)}{\bar{N}\bar{V}} \tag{27}$$

$$s_{luc} = A_{luc}\sqrt{\left(\frac{s_{\gamma}}{\gamma}\right)^2 + \frac{s_{cs}{}^2 + s_{\text{sup}}{}^2}{\left(l_{cs} - l_{\text{sup}}\right)^2} + \left(\frac{s_{\bar{N}}}{\bar{N}}\right)^2 + \left(\frac{s_{\bar{V}}}{\bar{V}}\right)^2} \tag{28}$$

s_{luc} is plotted in Figure 2d as the horizontal error bars.

Converting the QUEEN ratio values to ATP concentration in single cell measurements

Some of the additional information concerning the single-cell QUEEN signal acquisition and conversion into ATP concentration are described here.

In the cell suspension measurements (Fig. 2b, d), the autofluorescence of the cell suspension was measured separately using non-fluorescent cells and was subtracted from the total fluorescence intensity (see above). In contrast, when individual non-fluorescent cells (transformed with empty pRSET B vector) were observed under the microscope, the intensity of autofluorescence was very low. Hence, we did not subtract autofluorescence in the single-cell measurements, and instead, decided to use the cells with a fluorescence intensity above a specific threshold for statistical analysis.

In Supplementary Figure S6, the 405ex/480ex ratios of cells grown in continuous culture condition are plotted against fluorescence intensity. We only used cells that had a fluorescence intensity of over 40 arbitrary units in both channels. This threshold was chosen so as to include as many data points as possible, and at the same time, to minimize the effects of experimental noise and cell autofluorescence. Changing the threshold level around this value only slightly affected the SD of the data sets, and thus, we consider experimental noise and cell autofluorescence to be minimized.

The conversion of the QUEEN-2m excitation ratio to ATP concentration was performed using the ATP response curve of purified QUEEN-2m measured under the microscope (Fig. 4c, black line). This curve was corrected for slight signal difference of in vitro and in vivo as follows. The in vitro ratio data from the microscopic measurement was multiplied by the correction factor $C(x)$, and then the points were fitted with a Hill equation to obtain the corrected in vivo response curve (Fig. 4c, red line). The correction factor $C(x)$ was defined using the cell suspension measurement results (Fig. 2d) as follows,

$$C(x) = \frac{Z_{vivo}(x)}{Z_{vitro}(x)} \;\left(0 \le x \le 4\right),\; C(x) = 1 \;\left(x > 4\right) \tag{29}$$

Here, x is the ATP concentration (mM), $Z_{vitro}(x)$ is the 400ex/494ex ratio of purified QUEEN-2m (Fig. 2d, black line), and $Z_{vivo}(x)$ is the ratio of in vivo QUEEN-2m (Fig. 2d, green line). Since the in vivo data matched the in vitro data at an ATP concentration of approximately 4 mM, and in vivo data were not available for ATP concentration greater than this, $C(x)$ was set to 1 for ATP concentration above 4 mM. Although this may compromise the precision of QUEEN-2m at high ATP levels, in any case, the precision of QUEEN-2m is intrinsically inferior at high [ATP] (see below).

From this curve, the ATP concentration corresponding to the ratio value obtained was calculated (Supplementary Fig. S9a). Since the obtained ratio has a 5.82% error, the ATP value calculated also has an error, shown as error bars in

Supplementary Figure S9a. It must be noted that the law of error propagation assumes a symmetrical error around the true value of the 405ex/480ex ratio, and therefore, the error bars are symmetrical. In reality, the propagated error in ATP concentration estimation is asymmetrical in the high or low ratio regions because of the sigmoidal shape of the Hill curve. In Supplementary Figure S9b, relative error (error bar size divided by the estimated ATP value) is plotted against ATP concentration. The relative error is <20% at 0.40–7.4 mM ATP and <15% at 1.6–4.7 mM ATP. Thus, the response of QUEEN-2m to ATP change is the most sensitive in the range of approximately 1–5 mM.

The ATP concentration of cells grown in continuous culture (Fig. 4d) was calculated from the obtained distribution of the 405ex/480ex ratios (Fig. 4b). We saw no correlation between signal intensity and ratio value (Supplementary Fig. S6c). Hence, QUEEN expression level might not be correlated with the QUEEN ratio or ATP concentration.

Deconvolution of noise in the pH distribution measurement

The SD of the observed ratiometric pHluorin ratio distribution (σ_{Obs}) is larger than the SD of the true ratio (σ_T) due to stochastic experimental noise. If the SD of the noise (σ_{EN}) is known, the true SD of the major peak can be calculated as follows. Let the true ratio be x, which is distributed as $N(\langle x \rangle, \sigma_T)$, where $\langle x \rangle$ is the mean of x and,

$$\left\langle (x - \langle x \rangle)^2 \right\rangle = \sigma_T{}^2 \tag{30}$$

Let y be the observed ratio, which is a sum of the true signal and the experimental noise, e,

$$y = x + e \tag{31}$$

$$\langle e \rangle = 0 \tag{32}$$

$$\langle e^2 \rangle = \sigma_{EN}{}^2 \tag{33}$$

The mean and variance of y are then,

$$\langle y \rangle = \langle x + e \rangle = \langle x \rangle + \langle e \rangle = \langle x \rangle \tag{34}$$

$$\left\langle (y - \langle y \rangle)^2 \right\rangle = \sigma_{Obs}{}^2 = \left\langle (x - \langle x \rangle)^2 \right\rangle + \left\langle 2e(x - \langle x \rangle) \right\rangle + \langle e^2 \rangle = \sigma_T{}^2 + \sigma_{EN}{}^2 \tag{35}$$

where it is assumed that the signal x and the noise e are independent, so that

$$\left\langle 2e(x - \langle x \rangle) \right\rangle = 2\langle e \rangle \left\langle (x - \langle x \rangle) \right\rangle = 0. \tag{36}$$

The major peak in Figure 3f is fitted with a normal distribution, $N(1.90, 0.117)$, hence $\sigma_{Obs} = 0.117$. When the ΔpH was collapsed and extracellular pH was set to pH 8, the ratio distribution was fitted by $N(1.86, 0.089)$ (Fig. 3e). Accordingly, it can be assumed that $\sigma_{EN} = 0.089$. Using Eq. (35), $\sigma_T = 0.076$. Consequently, the true distribution of the major peak is estimated as $N(1.90, 0.076)$, corresponding to a pH value of 7.98 ± 0.18 (mean ± SD).

Calculation of the error bar size in the histogram

The error bars in the ATP concentration histogram of single-cell measurements were calculated by bootstrapping. First, we repeatedly re-sampled the obtained data sets 1000 times. That is, when we obtained a total of n ratio data points from the experiment, the ith ($0 \le i \le n$) ratio data point (z_i) was re-sampled by adding computer-generated noise ($e_{i,j}$) to each data point in the jth ($0 \le j \le 1000$) re-sampling.

$$u_{i,j} = z_i + e_{i,j} \quad (37)$$

Here, $u_{i,j}$ is the re-sampled ratio value. $e_{i,j}$ is defined to be normally distributed $N(0, z_i * 0.00582)$ to match the experimental error of the system. Next, the re-sampled ratio was converted to re-sampled ATP values ($v_{i,j}$) using the inverse Hill equation function $[h^{-1}(z)]$.

$$v_{i,j} = h^{-1}(z_{i,j}) \quad (38)$$

Then, for each jth sampling, the histogram of re-sampled ATP values was made. In the jth histogram, the number of data points in the kth bin $(0 \le k \le 40)$ was $a_{j,k}$. For each bin, the standard deviation was calculated from the total 1000 values of $a_{j,k}$. The obtained standard deviation is plotted in Figure 4d as the error bars.

Estimation of the true statistical parameter values

The statistical parameters (mean, SD and skewness) of the ATP concentration ([ATP]), corrected for experimental error in single-cell measurements, were evaluated by using the numerical estimator developed recently[36]. In this method, the estimator, which is a function of the observed fluorescence ratio and should be close to the real [ATP], is constructed by numerically minimizing the weighted sum of the bias and the risk, integrated over the relevant [ATP] range. The bias parameter b, which corresponds to the relative weight of the bias and the risk, was taken to be the inverse of the number of cells in each data set, following the discussion in Appendix B of Ref. 36. The weights for the integration over the fluorescence ratio were set by the distribution of the observed fluorescence ratio. More precisely, we put the grid points for the summation in Eq. (6) of Ref. 36 at all the observed values of the fluorescence ratio, each weighted by one ($w_i = 1$). The likelihood function, which is a probability distribution function for the observed fluorescence ratio at a given value of the real fluorescence ratio, was taken as the normal distribution centered at the real fluorescence ratio, with the 5.82% relative standard deviation from the calibration experiment described in Supplementary Methods. The target function (denoted g in Ref. 36) was set to be the real concentration, and its square or cube was given as a function of the real fluorescence ratio by using the Hill equation. Estimators for [ATP] and its square or cube was then applied to the value of the fluorescence ratio observed for each single cell. The estimated values were then averaged to obtain the first to third moments and the CV of the distribution of the real [ATP].

To check the validity of the above estimation, we performed the following simulation. A data set consisting of 100 cells was simulated by drawing 100 samples from the normal distribution $N(\langle x \rangle, \sigma_{ATP})$, where the average [ATP] $\langle x \rangle$ was set to $\langle x \rangle$ = 4 mM, which is in the range of the observed values in the present experiment, and the standard deviation, σ_{ATP}, was fixed at a specific value in the range of 0.1–1.0 mM, (the horizontal axis of Supplementary Fig. S10). The observed fluorescence ratios were then generated by a normal distribution centered at the true ratio

calculated from the Hill equation and with a relative standard deviation of 5.82%. This simulated data set was then subject to the analysis described above, and the standard deviation of the underlying [ATP] was estimated. The standard deviation was also estimated by a naïve estimator, which is obtained by simply substituting the observed ratio into the Hill equation. The same simulation was repeated 40 times for each value of σ_{ATP} in order to obtain statistics of the estimated values. Supplementary Figure S10 shows the estimated standard deviations as functions of the true standard deviations, σ_{ATP}. The naïve estimator overestimates the standard deviation in the whole range plotted in the figure. This is because the variance of the concentration estimated from the naïve estimator includes not only the variance of the underlying concentration but also the scattering due to measurement errors. In contrast, the results of the unbiased estimator are almost the same as the true values of the standard deviation.

Supplementary References

24 Kato-Yamada, Y. & Yoshida, M. Isolated epsilon subunit of thermophilic F1-ATPase binds ATP. *J. Biol. Chem.* **278**, 36013-36016, (2003).

25 Tran, Q. H. & Unden, G. Changes in the proton potential and the cellular energetics of Escherichia coli during growth by aerobic and anaerobic respiration or by fermentation. *Eur J Biochem* **251**, 538-543 (1998).

26 Bennett, B. D., Yuan, J., Kimball, E. H. & Rabinowitz, J. D. Absolute quantitation of intracellular metabolite concentrations by an isotope ratio-based approach. *Nat. Protoc.* **3**, 1299-1311, (2008).

27 Miyawaki, A. Visualization of the spatial and temporal dynamics of intracellular signaling. *Dev. Cell* **4**, 295-305, (2003).

28 Remington, S. J. Fluorescent proteins: maturation, photochemistry and photophysics. *Curr. Opin. Struct. Biol.* **16**, 714-721, (2006).

29 Iizuka, R., Yamagishi-Shirasaki, M. & Funatsu, T. Kinetic study of de novo chromophore maturation of fluorescent proteins. *Anal. Biochem.* **414**, 173-178, (2011).

30 Suskiewicz, M. J., Sussman, J. L., Silman, I. & Shaul, Y. Context-dependent resistance to proteolysis of intrinsically disordered proteins. *Protein Sci.* **20**, 1285-1297, (2011).

31 Wang, Q., Shui, B., Kotlikoff, M. I. & Sondermann, H. Structural basis for calcium sensing by GCaMP2. *Structure* **16**, 1817-1827, (2008).

32 Yu, J., Xiao, J., Ren, X., Lao, K. & Xie, X. S. Probing gene expression in live cells, one protein molecule at a time. *Science* **311**, 1600-1603, (2006).

33 Stouthamer, A. H. & Bettenhaussen, C. Utilization of energy for growth and maintenance in continuous and batch cultures of microorganisms. A reevaluation of the method for the determination of ATP production by measuring molar growth yields. *Biochim. Biophys. Acta* **301**, 53-70, (1973).

34 Stephanopoulos, G., Aristidou, A. A. & Nielsen, J. H. *Metabolic engineering : principles and methodologies*. (Academic Press, 1998).

35 Hempfling, W. P. & Mainzer, S. E. Effects of varying the carbon source limiting growth on yield and maintenance characteristics of Escherichia coli in continuous culture. *J. Bacteriol.* **123**, 1076-1087, (1975).

36 Kawai, S. *et al.* Numerical construction of estimators for single-molecule fluorescence measurements. *J. Phys. Chem. B* **117**, 8061-8074, (2013).

Supplementary Figure Legends

Supplementary Figure S1

The effect of ADP to the signal of purified QUEENs. (**a**, **d**) Response of 400ex/494ex ratio to ATP. Solid lines are the results of fitting to Hill equations with either

cooperativity = 1 (**a**) or cooperativity = 2 (**d**). (**b**, **e**) Experimental results of signal change when ADP, which is contaminated with trace amounts of ATP, was added to the solution. The horizontal axis shows the concentration of apparent ADP, assuming that pure ADP was added to the solution. Different markers show different set of experiments with various initial concentrations of ATP. Lines are the results of fitting to Eq. (7) in (**b**) or Eq. (9) in (**e**). (**c**, **f**) Calculated 400ex/494ex ratio values plotted against ADP concentration. The calculations are based on parameters obtained by fitting in (**b**) and (**e**). Each line shows different true ATP values. For reference, the 5.82% error in ratio values for the single cell microscope measurements (See Fig. 3 and text) were plotted together as the error bars. (**g**) Apparent ATP values when ADP exists in the solution. The 400ex/494ex values in (**f**) were converted to ATP values. The error bar lengths were also converted using the law of error propagation. (**a**-**c**) QUEEN-7μ, (**d**-**g**) QUEEN-2m. Measurements were done in Buffer C at 25°C. For the experiments in this figure, $MgSO_4$, instead of $MgCl_2$, was mixed with the nucleotides to make ATP-Mg^{2+} or ADP-Mg^{2+}. In addition, spectrofluorometer FP-6200 (JASCO) was used for the measurement.

Supplementary Figure S2

Properties of QUEEN-2m measured in vitro. (**a**) Emission spectra of QUEEN-2m. ATP concentration was 0 (blue), 1.9 (green), and 9.4 (magenta) mM. The excitation wavelength was 400 nm (broken lines) or 494 nm (solid lines). (**b**) Response of QUEEN-2m to ATP concentration at different Mg^{2+} ion concentrations. The buffer composition was the same as buffer C except for the different Mg^{2+} (as $MgCl_2$) concentrations. (**c**) Response speed of QUEEN-2m.

Supplementary Figure S3

Simulation result of the maturation and degradation kinetics of ATeam when the growth rate is decreased. (**a**) Time course of the concentration of different ATeam states. At t = 5, the growth rate changed from μ_1 = 0.25 to μ_2 = 0.10. Blue line: fully mature FRET-type indicator. Red broken line: half-mature FRET-type indicator. Green dot-dashed line: half-degraded FRET-type indicator. The parameters used to generate these data were a = 100, k_1 = 1.5, k_2 = 0.05, k_3 = 0.05. (**b**) Time course of yellow fluorescent protein (YFP)/cyan fluorescent protein (CFP) ratio for the simulation result in (**a**), assuming that the ATeam was AT3.10 and that changes in ATP concentration could be ignored. See Supplementary Note for the details.

Supplementary Figure S4

QUEEN-2m (**a**) and $AT1.03^{YEMK}$ (**b**) signals in cell suspension measurement when ATP is added to the extracellular solution. The broken gray lines represent the in vitro response curve of each indicator to the ATP concentration for reference.

Supplementary Figure S5

Procedures for cell fixation onto the cover slip using a thin agarose film. (**a**) Preparation of the agarose film. Cover slips and PDMS sheets (0.5 mm-thick) were assembled as shown, and melted agarose was flowed into the designated space. (**b**) Placing the agarose film on the bacteria cells.

Supplementary Figure S6

The distribution of the 405ex/480ex values of QUEEN-7μ, QUEEN-NA and QUEEN-2m cells. (**a**–**c**) The 405ex/480ex ratio of QUEEN-7μ (**a**), QUEEN-NA (**b**) and QUEEN-2m (**c**) cells, plotted against the signal intensity at 480-nm excitation. Cells were sampled at the steady state in continuous culture. Gray data points indicate the cells that are not included in the statistical analysis due to low signal intensity (see Supplementary Methods).

Supplementary Figure S7

The chemostat system used to grow cells in a continuous culture condition. Three reactors are shown in the illustration, although the number of reactors can be modified.

Supplementary Figure S8

E. coli cell volume estimation. (**a**) Representative image of cells stained with ATTO655-NHS ester for volume estimation. The position of the membrane is visualized as a fluorescent layer. (**b**) Fluorescence intensity along the line shown in (**a**). There are two peaks at the position of the membrane. The distance between the peaks is assumed to be the length of the short axis of this cell. (**c**) Histograms of the measured cell sizes for QUEEN-2m (top), and AT1.03^{YEMK} (bottom) cells.

Supplementary Figure S9

The precision of ATP measurement by QUEEN-2m, considering the noise level of 5.82% in the 405ex/480ex ratio value measured under the microscope. (**a**) ATP concentration plotted against the 405ex/480ex ratio. The error bars show the standard error of the calculated ATP concentration, assuming that 5.82% noise is included in the value of the ratio. The error was calculated using the law of error propagation. The error bars are plotted at 0.05 ratio value intervals. (**b**) The relative standard error of ATP concentration, i.e., standard error in (**a**) divided by the corresponding ATP concentration value, plotted against ATP concentration.

Supplementary Figure S10

Computer simulation to check the validity of the standard deviation estimation methods in the presence of experimental error. Squares: standard deviations estimated according to the (almost) unbiased estimator of Ref. 36, which is mainly used in this study. Circles: standard deviations estimated according to the naïve

estimator, which is obtained by simply substituting the observed ratio into the Hill equation. See Supplementary Methods for additional details.

Supplementary Figures

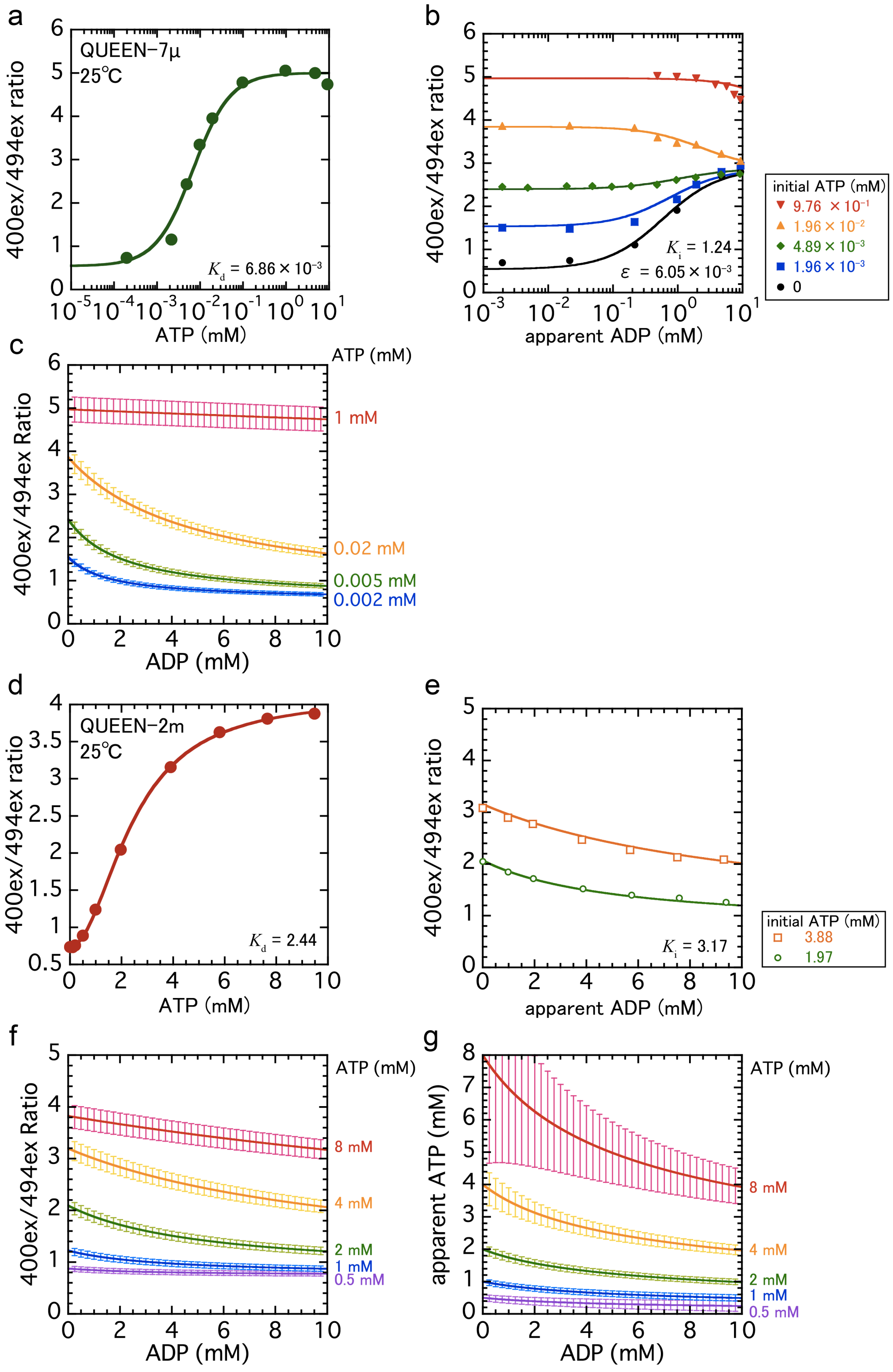

Supplementary Fig. S1

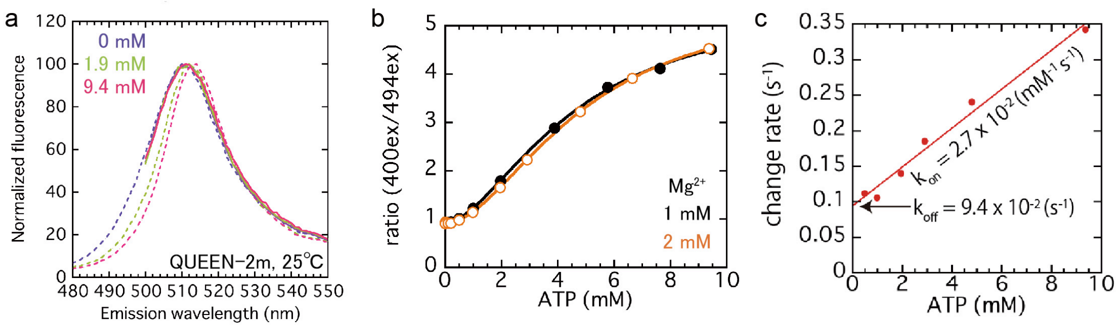

Supplementary Figure S2

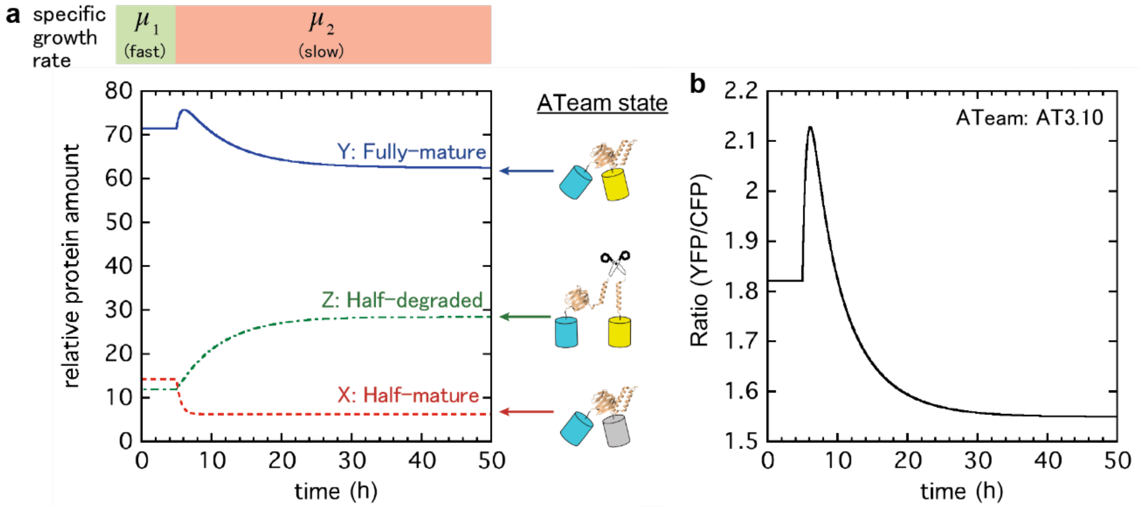

Supplementary Figure S3

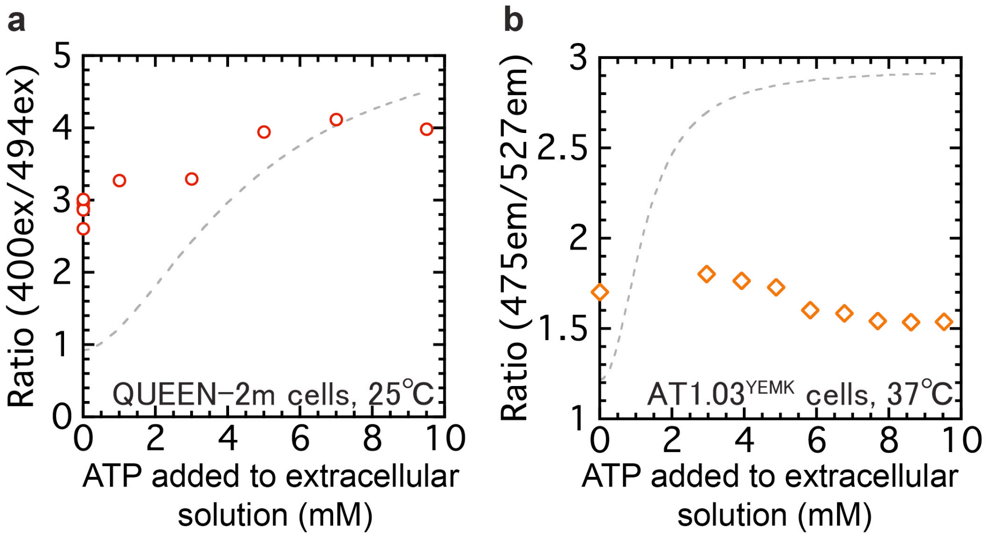

Supplementary Figure S4

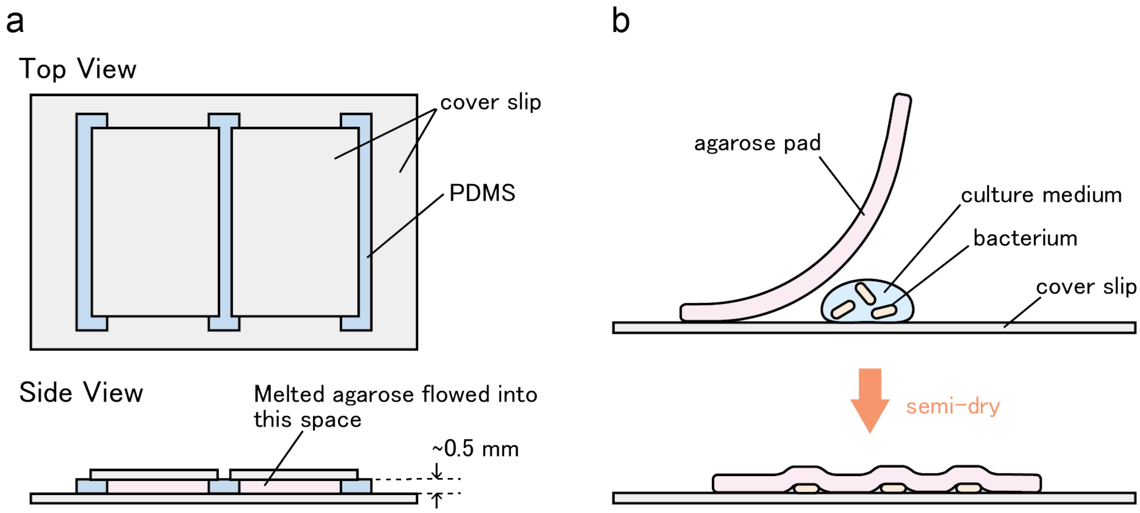

Supplementary Figure S5

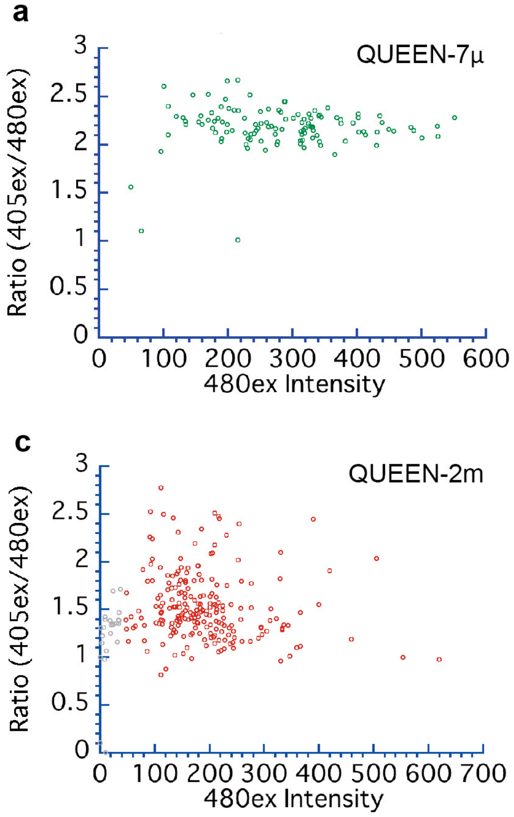

Supplementary Figure S6

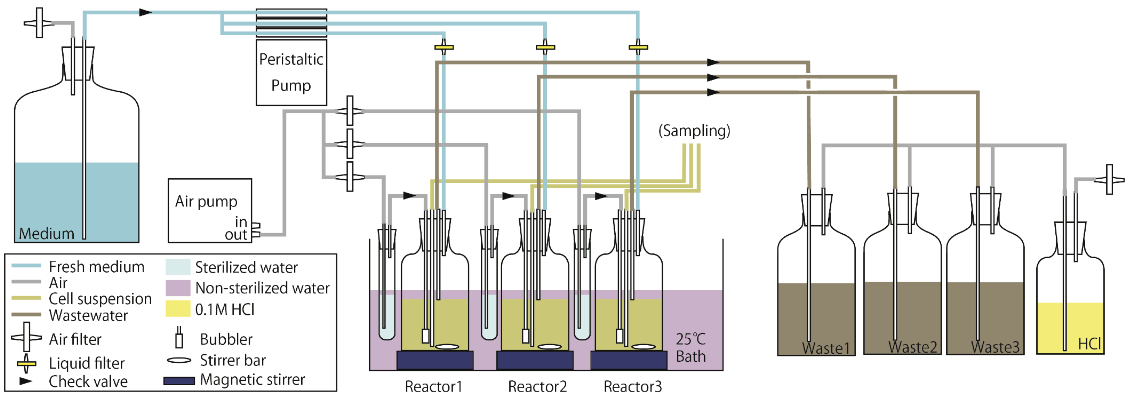

Supplementary Figure S7

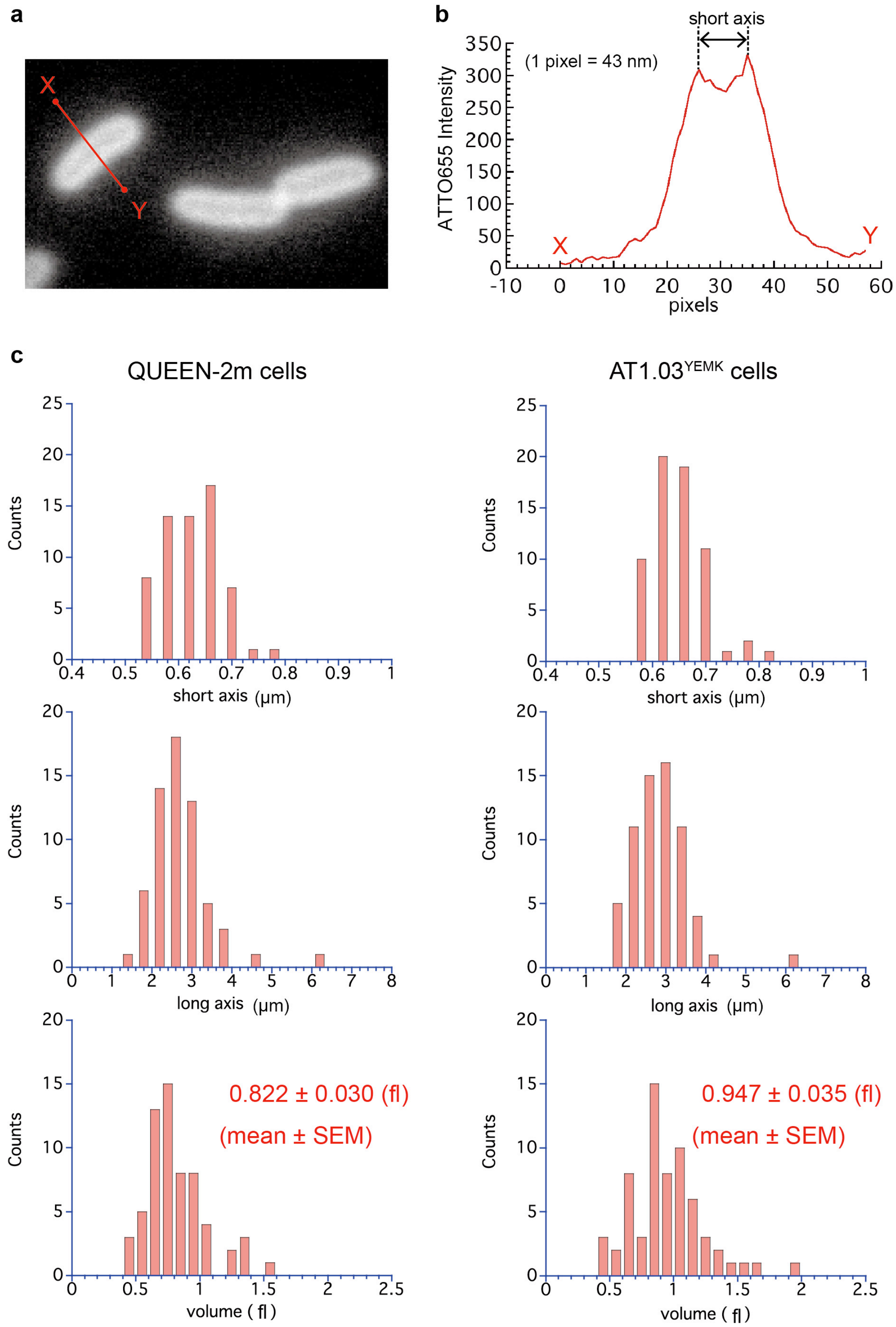

Supplementary Figure S8

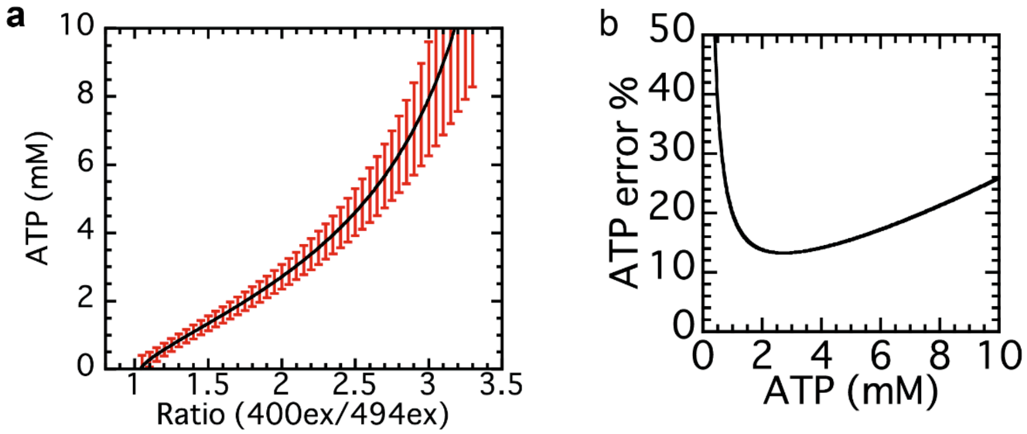

Supplementary Figure S9

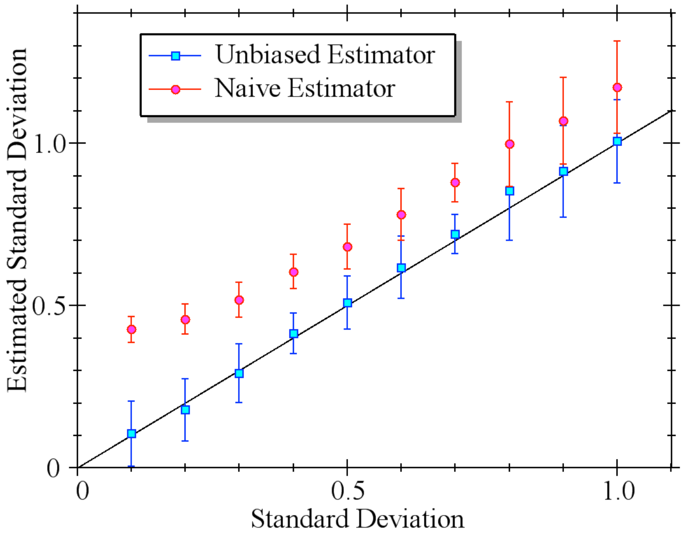

Supplementary Figure S10
